# Overexpression of jojoba wax ester synthase in poplar increases foliar lipid accumulation, alters stomatal conductance, and increases water use efficiency

**DOI:** 10.1111/plb.70056

**Published:** 2025-05-27

**Authors:** A. Amirkhosravi, G.‐J. Strijkstra, A. Keyl, F. Häffner, U. Lipka, C. Herrfurth, I. Feussner, A. Polle

**Affiliations:** ^1^ Forest Botany and Tree Physiology, Büsgen‐Institute and Göttingen Center for Molecular Biosciences (GZMB), Büsgenweg 2 University of Göttingen Göttingen Germany; ^2^ Department of Plant Biochemistry, Albrecht‐von‐Haller‐Institute of Plant Sciences and Göttingen Center for Molecular Biosciences (GZMB), Justus‐von‐Liebig‐Weg 11 University of Göttingen Göttingen Germany; ^3^ Service Unit for Metabolomics and Lipidomics, Göttingen Center for Molecular Biosciences (GZMB), Justus‐von‐Liebig‐Weg 11 University of Göttingen Göttingen Germany

**Keywords:** Cuticle wax, drought stress, jojoba, occluded stomata, photosynthesis, wax biosynthesis

## Abstract

- Poplars are important fast‐growing, water‐spending biomass tree crops. Here, we targeted the wax biosynthesis pathway of *Populus* × *canescens* by overexpressing jojoba (*Simmondsia chinensis*) wax ester synthase (*Sc*WS) for the production of lipids.
- We found that *ScWS* overexpression caused accumulation of lipid droplets in leaf cells but did not increase wax load on the leaf surface. However, the stomata had a high fraction of closed or semi‐closed guard cells with aberrant lipid accumulation. This phenotype was accompanied by suppression of *OCCLUDED STOMATAL PORE 1* (*OSP1*) and decreased stomatal conductance in the *ScWS*‐expressing poplars.
- During short‐ and long‐term drought scenarios under greenhouse and outdoor conditions, the Sc*WS* lines had increased water use efficiencies, leading to a water‐saving phenotype and delays in drought stress. In the *Sc*WS poplars, photosynthesis was reduced under high, but not under low light intensities. Biomass production of Sc*WS* lines was unaffected in short‐term experiments but dropped below that of wild‐type poplars at the end of two field seasons, indicating a long‐term growth trade‐off.
- Our results open new opportunities for the production of lipids in more water‐efficient poplars.

## Graphical abstract

Overexpression of jojoba wax ester synthase in poplar causes accumulation of intracellular lipids in leaves and reduced stomatal conductance with higher water‐use efficiencies in the transgenic lines.

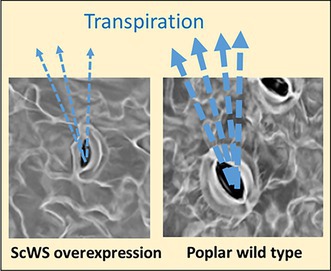

## INTRODUCTION

Wax esters are found in a wide range of organisms, including algae, animals, microbes, and plants (Domergue & Miklaszewska 2022). They are hydrophobic compounds, composed of a fatty alcohol esterified to a fatty acid moiety. In many plant species, wax esters are important components of coatings on leaves, stems, and pollen and protect the aboveground organs against desiccation, radiation, and feeding insects (Lewandowska *et al*. 2020). In addition, waxes have industrial applications, e.g., as lubricants and in cosmetics and pharmaceuticals, but they occur naturally, in most species, only in low quantities (Domergue & Miklaszewska 2022). Among the few exceptions is *Simmondsia chinensis* (Jojoba), which accumulates wax esters, instead of triacylglycerols, in seeds as storage lipids (Lardizabal *et al*. 2000).

In plants, wax esters commonly occur as protective components on the outer hydrophobic leaf surface, the cuticle, in a mixture with other aliphatics as so‐called extracuticular waxes (Samuels *et al*. 2008; Zeisler‐Diehl *et al*. 2020). Cuticular waxes are a mixture of monomeric very long‐chain (VLC) aliphatics, composed of fatty acids, fatty aldehydes, primary and secondary fatty alcohols, alkenes, alkanes, and esters of fatty acids and fatty alcohols (Samuels *et al*. 2008; Zeisler‐Diehl *et al*. 2020). They block cuticular water loss and, thus, are essential to protect plants from uncontrolled evapotranspiration (Jetter & Riederer 2016; Seufert *et al*. 2022).

Plants have two different enzyme families for wax ester biosynthesis, prokaryotic bifunctional wax ester synthase/acyl‐CoA diacylglycerol acyltransferases (WSDs) and eukaryotic mono‐functional wax ester synthases (WSs) (Cheng *et al*. 2022). The formation of waxes often starts with the elongation of C16 and C18 acyl‐CoAs to VLCFAs, which contain 20 or more carbons (Samuels *et al*. 2008; Hegebarth *et al*. 2017). Elongation takes place in the ER membrane. The first step is mediated by 3‐ketoacyl‐CoA synthases (KCS), which are members of the FATTY ACID ELONGASE (FAE) enzyme complex. Some KCSs belong to the group of so‐called *CER (ECERIFERUM*) genes (Haslam & Kunst 2013). Mutations in *CER* genes cause disturbances in wax composition (Koornneef *et al*. 1989). After elongation, VLC acyl‐CoAs can be either incorporated into glycerol‐ or sphingolipids or undergo further modifications via the alcohol‐ or alkane‐forming pathways (Lewandowska *et al*. 2020). In the alcohol‐forming pathway of *Arabidopsis thaliana*, VLC acyl‐CoAs are reduced to primary alcohols by fatty acyl‐CoA reductase 3 (*At*FAR3/ *At*CER4) (Rowland *et al*. 2006). In the case of cuticular waxes, a portion of the primary alcohols can be esterified with acyl‐CoAs (mainly C16) by *At*WSD1, which is localized in the ER of epidermal cells (Li *et al*. 2008). Further studies revealed that overexpression of *At*WSD1 promotes drought tolerance of *A. thaliana* (Patwari *et al*. 2019). Moreover, heterologous *AtWSD1* expression in *Camelina sativa* caused a massive increase in the thickness of the cuticle wax layer and mediated enhanced resistance to various osmotic stresses (Abdullah *et al*. 2021).

Enzymes of the plant WS family also reside in the ER membrane, but they form wax esters for endogenous storage in lipid droplets. The WS from the drought‐tolerant desert species jojoba [*Simmondsia chinensis* (*Sc*WS)] was the first enzyme identified from this subfamily (Lardizabal *et al*. 2000). *Sc*WS uses monoenoic aliphatic chains to form wax esters of high stability (Gad *et al*. 2021), whose properties are of interest in biorefinery (Iven *et al*. 2016; Yu *et al*. 2018; Alotaibi *et al*. 2020). Although *A. thaliana* contains at least 12 homologues of WS enzymes in its genome, their function remains enigmatic until today. Only one, At5g55380, is present in extra‐plastidial membranes and strongly expressed in vegetative tissues at the bolting stage (Cheng *et al*. 2022). Moreover, it produced wax esters when expressed under a seed‐specific promoter (Cheng *et al*. 2022). However, it is still an open question whether wax ester biosynthesis by WS enzymes can be manipulated in leaves, and whether this interferes with the composition and function of the cuticle. This question is pertinent, since the composition of the cuticle varies with environmental stress, constitutes a barrier against invading pathogens (Lewandowska *et al*. 2020; Shaheenuzzamn *et al*. 2021), and is decisive in preventing water loss (Grünhofer *et al*. 2022; Seufert *et al*. 2022). Furthermore, modifications in the biosynthesis of cuticular components might affect stomatal development (Wang & Chang 2024) and, thus, may have consequences for plant water‐use efficiency.

Here, we used poplar (*Populus* × *canescens*) trees for heterologous expression of *Sc*WS. Poplars are economically important biomass crops because of their fast growth (Taylor *et al*. 2019). The productivity of poplars is susceptible to water scarcity (Polle *et al*. 2019). We expected that a constitutive expression of jojoba wax ester synthase (*Sc*WS) would increase the wax ester content in leaves, thereby shifting the balance of lipids away from cuticular wax production, with potentially negative effects on the barrier properties of the cuticle. Alternatively, we considered that a potential increase towards wax biosynthesis in the transgenic poplars might augment the cuticular wax load or composition, thereby reducing evaporation of water and improving fitness of the transgenic plants under drought. Since poplars are highly water‐demanding species, we reasoned that any change that affects their water economy might alter their ability to acclimate to changing environmental conditions. Therefore, our study had two goals: (1) to generate poplars with increased wax ester content, and (2) to investigate whether this modification affected the water‐use efficiency and stress resistance of the transgenic lines. For this purpose, we tested the performance of wild‐type (WT) and *Sc*WS poplars under greenhouse conditions and in small plantation‐like settings in outdoor conditions for two growth seasons under well‐irrigated or drought‐stress conditions. We show that expression of *Sc*WS increases the cellular lipid content and has moderate effects on wax composition, but does not increase the overall cuticular wax load of the leaf. Unexpectedly, guard cells of the ScWS poplars exhibited aberrant lipid accumulation, accompanied by partial stomatal closure. Consequently, *Sc*WS poplars have lower stomatal conductance and higher water‐use efficiency. Together our data show a tight connection between endogenous lipid biosynthesis and stomatal features.

## MATERIAL AND METHODS

### Poplar cultivation and transformation

Stock cultures of *Populus* × *canescens*, a hybrid of *P*. *alba* × *P*. *tremula* (clone INRA 717‐1B4), were micropropagated under sterile conditions (Müller *et al*. 2013) and used for *Agrobacterium*‐mediated shoot transformation following protocols of Bruegmann *et al*. (2019) and Muhr *et al*. (2016). The details are reported in Supplementary Methods S1. Regenerating plantlets from independent transformation events were tested for the presence of the target gene, *ScWS*, via Sanger sequencing (Microsynth Seqlab, Göttingen, Germany). Successfully transformed lines carrying the *p35S::ScWS* construct were named *ScWS1*, *ScWS2*… *ScWSn*. For the experiments, we used *ScWS1* to *ScWS4*.

### Short‐term, long‐term, and outdoor growth phenotyping and drought experiments

The WT and *ScWS* lines from sterile stock cultures were multiplied through stem microcuttings and cultured on ½ MS medium (Murashige & Skoog 1962) with a 16 h light/8 h dark photoperiod until the plantlets were well rooted. Approximately 6‐week‐old plantlets were directly planted into soil: two parts (*v/v*) Fruhstorfer Erde Type N; (Hawite Gruppe, Vechta, Germany), eight parts coarse sand (Ø 0.71–1.25 mm; Melo, Göttingen, Germany), and two parts fine sand (Ø 0.4–0.8 mm; Melo) and acclimated for about 2 weeks to greenhouse conditions. For this purpose, the plants were initially covered with a beaker, which was gradually removed (Müller *et al*. 2013). For short‐term experiments, 3‐L pots, and for long‐term experiments, 7‐L pots were used. For the outdoor experiments, a caged area (Göttingen, Germany, 51.55739° N, 9.95857° E, 153 m a.s.l.; Yu *et al*. 2019), with large containers (3 m × 3 m × 0.5 m) filled with a mixture of sand and compost (Vogteier Erdenwerk, Niederdorla, Germany) was used. Potted plants were acclimated to greenhouse and then to outdoor conditions, before planting into the outdoor containers. To control water supply, the soil surface was covered with polyethylene foil and the plants were watered using a drip watering system programed and managed by an intelligent controller AQUA PRO Irrigation Controller (NETAFIM, Tel Aviv, Israel).

#### Short term experiment

We used three cabinets (temperature: 23–24°C, relative humidity 60%–70%). Ambient light was supplemented with additional illumination (3071/ 400 HI‐I; Adolf Schuch, Worms, Germany) to obtain a light period of 16 h and 150 μmol photons m^−2^ s^−1^ PAR at plant height (Yu *et al*. 2019). In each cabinet, 40 plants were randomly distributed. We used 24 plants of the lines *ScWS1*, *ScWS2*, *ScWS3*, *ScWS4* and the WT. The plants were grown for 2 additional months and used to measure stem height, stem diameter, and leaf number, once a week. For the drought treatment, plants (70 cm height) in each cabinet were divided into two groups, each containing 7 to 8 plants. The control group received 400 mL water day^−1^ and the drought‐stressed group, 100 mL. The soil water content was monitored (HH2 with ML2x sensor, Delta T Devices, Cambridge, UK) twice a day (08:00 to 08:30 h and 15:00 to 15:30 h) in the pots of all plants. If the soil water content fell below the threshold of 0.35 m^3^ m^−3^ (controls) or 0.09 m^3^ m^−3^ (severe drought), additional water was administered. After 1 week of drought treatment, the plants were harvested.

#### Long‐term experiment

Poplar WT and lines *ScWS1* and *ScWS2* were grown for 3 months under the same conditions as described for the short‐term experiment. Growth was recorded regularly. The plants (*n* = 8 per line and treatment) were exposed to drought stress by reducing water supply for 3 weeks to ca. 100 mL day^−1^ instead of 400 mL day^−1^ (controls), adjusting the amount of water to maintain target levels of soil moisture of 0.4 m^3^ m^−3^ and 0.1 m^3^ m^−3^ for well‐irrigated and drought‐stressed plants, respectively. After 3 weeks of drought treatment, the plants were harvested.

#### Outdoor experiment

In each container, 49 plants (7 × 7) were planted in October 2018 at a distance of 0.42 m × 0.42 m, resulting in 25 center and 24 border plants. The poplar lines were distributed so that all genotypes were mixed (Fig. S10, Planting scheme). The plants were watered for 10 min twice a day (08:00 h and 12:00 h). In the following year (2019), the plants were initially watered twice a day for 10 min and, from end of July until September, three times per day for 30 min, and then until the end of the season, three times per day for 10 min. Drought‐exposed plants were not watered from August to October. Soil humidity was measured weekly (HH2 with ML2x sensor). Air temperatures (°C) were monitored by a weather station next to the experiment. Means per season (data [°C] for year 1/year 2) were: winter 3.5/4.8, spring 9.1/8.8, summer: 18.7/17.9, fall: 10.3/10.4. Stem height and diameter were measured regularly. According to plantation practice, the poplars were cut back to the stool at the end of the growth phase in October 2019 by cutting the stem 10 cm above ground level. Leaves and wood biomass were separated, dried at 70°C and weighed. In the following year, resprouting and growth were observed. All plants were watered regularly by the irrigation system. The plants were harvested at the end of the growth season in October 2020.

### Harvest

Whole‐plant leaf and stem biomass were weighed (fresh and dry). Leaf area of selected leaves was determined by scanning (Image J, https://imagej.net/ImageJ). The area and weight of the selected leaves was used for upscaling to whole‐plant leaf area by multiplication with whole‐plant leaf biomass. Aliquots of fresh tissue (leaf, bark, wood) were immediately shock‐frozen in liquid nitrogen and stored at −80°C.

### Gas exchange

Gas exchange (photosynthesis, stomatal conductance, transpiration) was measured on fully expanded, light‐exposed leaves, usually leaf number 7 from the stem apex, with a multiphase Flash™ Fluorimeter (LI‐6800; LI‐COR, Lincoln, NE, USA). Daytime measurements were conducted between 10:00 h and 14:00 h with 800 μmol photons m^−2^ s^−1^ PAR, leaf temperature of 25–27°C, and ambient CO_2_ of 400 μmol mol^−1^. Instantaneous water‐use efficiency was determined as the ratio of net photosynthetic CO_2_ consumption to transpiration (Farquhar *et al*. 1989). Respiration, transpiration and stomatal conductance at night were measured between 02:00 h and 06:00 h in darkness. The measurements were conducted on *n* = 5 plants per line and treatment.

### Predawn leaf water potential

Predawn water potentials (SKPM 1400/40 pressure chamber; UP, Ibbenbüren, Germany) were measured on the petiole of detached leaves (Scholander *et al*. 1964) at night. Leaves from the same position of the stressed and control plants were used.

### Water loss assay

We used fully expanded leaves formed during drought and control leaves of the same age for the water loss assays. Ten leaf disks (Ø 14 mm) were punched out per leaf. The leaf disks floated on distilled water at 23 ± 0.5°C in darkness overnight. The water‐saturated leaf disks (SW) were surface‐dried, weighed, and exposed in an open glass Petri dish with the stomatal side on the glass surface. The weight of the disks was determined regularly for 5 h (WT_1_, WT_2_…, WT_5_). The leaf disks were oven‐dried at 80°C for 24 h and weighed (DW). Relative water loss was determined as:

$$\text{Water loss}\;\left(\%\right)=\left(\mathrm{SW}-\mathrm{WT}\right)\times 100/\left(\mathrm{SW}-\mathrm{DW}\right)$$

The relative water loss (%) was plotted against time, and used to determine the slope of a linear regression from 1 h to 5 h exposure. We did not include the start (0 h to 1 h) because differences in stomatal conductance and non‐specific water loss via the injured edges affected water loss in the initial phase. Mean slopes (% h^−1^) were determined for four independent biological replicates per treatment and line. Several independent repeated experiments were conducted with similar results.

### Scanning electron microscopy (SEM) of leaf surfaces and stomatal analyses

Fresh leaves of greenhouse‐ and field‐grown WT and *ScWS* poplars were used after sputtering with gold for SEM analyses as described in Dreischhoff *et al*. (2023). The details are presented in Supplementary Methods S1. A minimum of three independent biological samples were analysed per treatment and line. We used scans of the abaxial side of each leaf. Two areas were chosen per sample and the numbers of stomata and epidermal cells counted. The counts were used to calculate stomatal density (number of stomata mm^−2^) and stomatal index (number of stomata/number epidermal cells). The inner lengths of 75 stomata per sample were measured.

### Transmission electron microscopy (TEM) of leaf cross‐sections

Small leaf segments (max. 0.2 × 0.2 cm) were collected, fixed in glutaraldehyde (Science Services, München, Germany) overnight and post‐fixed in OsO_4_ (Science Services) at room temperature for 1 h. Then, the samples were dehydrated in an ethanol series and embedded in Araldite‐Epon resin according the manufacturer's instructions (Araldite 502/Embed 812 Kit; Science Services). Ultrathin cross‐sections (70 nm) were obtained with an Ultracut E microtome (Reichert‐Jung, Heidelberg, Germany) and a diamond knife. The sections were placed on Formvar‐coated nickel grits (Plano, Wetzlar, Germany) (Miklaszewska *et al*. 2023). After contrasting with UranyLess (Science Services) and 3% lead citrate (Science Services) following the manufacturer's instructions, pictures were captured by TEM at 80 kV using a Technai G2 Spirit (FEI company, Eindhoven, Netherlands).

### Confocal laser scanning microscopy (CLSM) of leaf sections

Fresh leaf samples were stained with LipidSpot‐488 (Lipid Droplet Stain, Biotium, Fremont, CA, USA) according to manufacturer's protocol with minor modifications: samples were incubated in darkness at 37°C for 10 min in a solution of 1 μL LipidSpot‐488 in 1000 μL phosphate buffer. The stained specimens were embedded on slides with 10 μL Roti embedding flour care (Carl Roth, Karlsruhe, Germany). Fluorescence was detected using a CLSM (TCS‐SP8; Leica, Wetzlar, Germany) at excitation wavelengths of 480–490 nm and emission wavelengths of 580–620 nm through the plane of the leaf (Fig. S5). Images were recorded at 100× magnification and used to count lipid droplets per area unit. Five biological replicates were analysed per line. Each biological replicate was the mean of two replicates per sample. Since the images revealed irregular stomatal shapes of ScWS lines, they were also used to characterize the stomatal phenotype. For this purpose, we classified the stomata as open, semi‐open, or occluded, and counted the number of stomata in each category.

### Cuticular wax analysis

For cuticular wax extraction, three leaf discs per plant of 14 mm in diameter with an area of 2.5 cm^2^ were collected, exposed for 30 s to chloroform containing *n*‐tetracosane as internal standard and further processed as previously described (Haslam & Kunst 2013). Derivatized samples were separated by gas chromatography and mass spectroscopy as described in detail in Supplementary Methods S1. The internal standard was used for the quantification of compounds. Very low amounts of odd number wax ester were not described because they could not be discerned from potential extractant contaminations.

### Thin layer chromatographic separation of total lipid extracts of *P.* × *canescens* leaves

Crude lipid extracts were prepared according to Iven *et al*. (2013) with minor modifications. Frozen leaf samples (−80°C) were milled and 100 mg used for extraction in chloroform:methanol (1:1, V/V). The lipid extracts were spotted on 0.25 × 20 × 20 cm F60 silica gel glass plates (Merck, Darmstadt, Germany) and separated by a mobile phase of hexane:diethyl ether:acetic acid (80:20:1). Wax ester and sterol ester were used as the reference compounds. The plates were sprayed with primuline dissolved in 80% acetone and viewed under UV light.

### RNA extraction, cDNA synthesis, and quantitative real time polymerase chain reaction (qRT‐PCR) of genes involved in wax biosynthesis

Frozen tissues were ground in a pre‐cooled ball mill (Retsch, Haan, Germany) to a fine powder. The frozen powder (150 mg) was used for total RNA extraction using the CTAB method (Chang *et al*. 1993). Genomic DNA was removed according to the manufacturer's instructions using the Turbo DNA‐free kit by treatment with 10 μg DNase (Turbo DNA‐free kit; Ambion, Austin, TX) at 37°C for 30 min. The integrity of the purified RNA (2.5 μg) was assessed by agarose gel electrophoresis (Fleige & Pfaffl 2006). DNase‐treated total RNA (5 μg) was used as starting material for double‐stranded cDNA synthesis using Oligo(dT)18 primer and RevertAid™ First Strand cDNA Synthesis Kit (Thermo Scientific, Sindelfingen, Germany) according to the manufacturer's protocol.

Quantitative Real‐time PCR (qRT‐PCR) was performed in 96‐well plates (qTOWER3 G touch; Analytik Jena, Jena, Germany). Primers for genes of the wax biosynthesis pathway were designed on the basis of *P. trichocarpa* and *P*. × *canescens* sequences (Mader *et al*. 2016) with the Perl Primer software v. 1.1.20 (Marshall 2004) (Table S1). The primer efficiency was controlled by running a dilution series. The qRT‐PCRs were carried out with following conditions: starting temperature 95°C for 2 min, then 45 cycles of 95°C for 10 s and 55°C for 20 s and at the end a melting curve analysis with measurements between 72 and 95°C. The 2^−ΔΔCt^ method was used to calculate relative expression of the genes using *PtrPPR_2* and *PtrRpp14* as reference (Livak & Schmittgen 2001).

### Analysis of abscisic acid (ABA)

We used the procedure described by Yu *et al*. (2021). After extraction from milled frozen leaf powder, phytohormones were separated by reverse‐phase chromatography on an ACQUITY UPLC® system (Waters, Milford, MA, USA) and analysed by nanoelectrospray (nanoESI) (TriVersa Nanomate®; Advion BioSciences, Ithaca, NY, USA) coupled with an AB Sciex 4000 QTRAP® tandem mass spectrometer (AB Sciex, Framingham, MA, USA).

### Statistical analyses

The Software R (v. 4.2.2, R Core Team, 2017), Origin 2020 (OriginLab®, Northhampton, MA, USA) and Statgraphics Centurion XVII (Statgraphics Technologies, The Plains, Virginia, USA) were used for statistical analysis. We tested normal distribution by inspecting residuals and homogeneity of variances using Levene's test. When the data were not normally distributed, we used log10 transformation to meet the statistical requirements. Data were analysed by ANOVA (line and drought as main factors) and a *post‐hoc* test to determine significant differences between means at *P* ≤ 0.05. If normal distribution and variance homogeneity were not achieved, non‐parametric methods and Kruskal‐Wallis test were applied. Data are shown as mean (± SE). The number individual biological replicates and the statistical tests are indicated in the figure legends.

## RESULTS

### Characterization of *Sc*WS‐expressing poplar lines reveals enhanced accumulation of lipid droplets in leaves

**Fig. 1:**
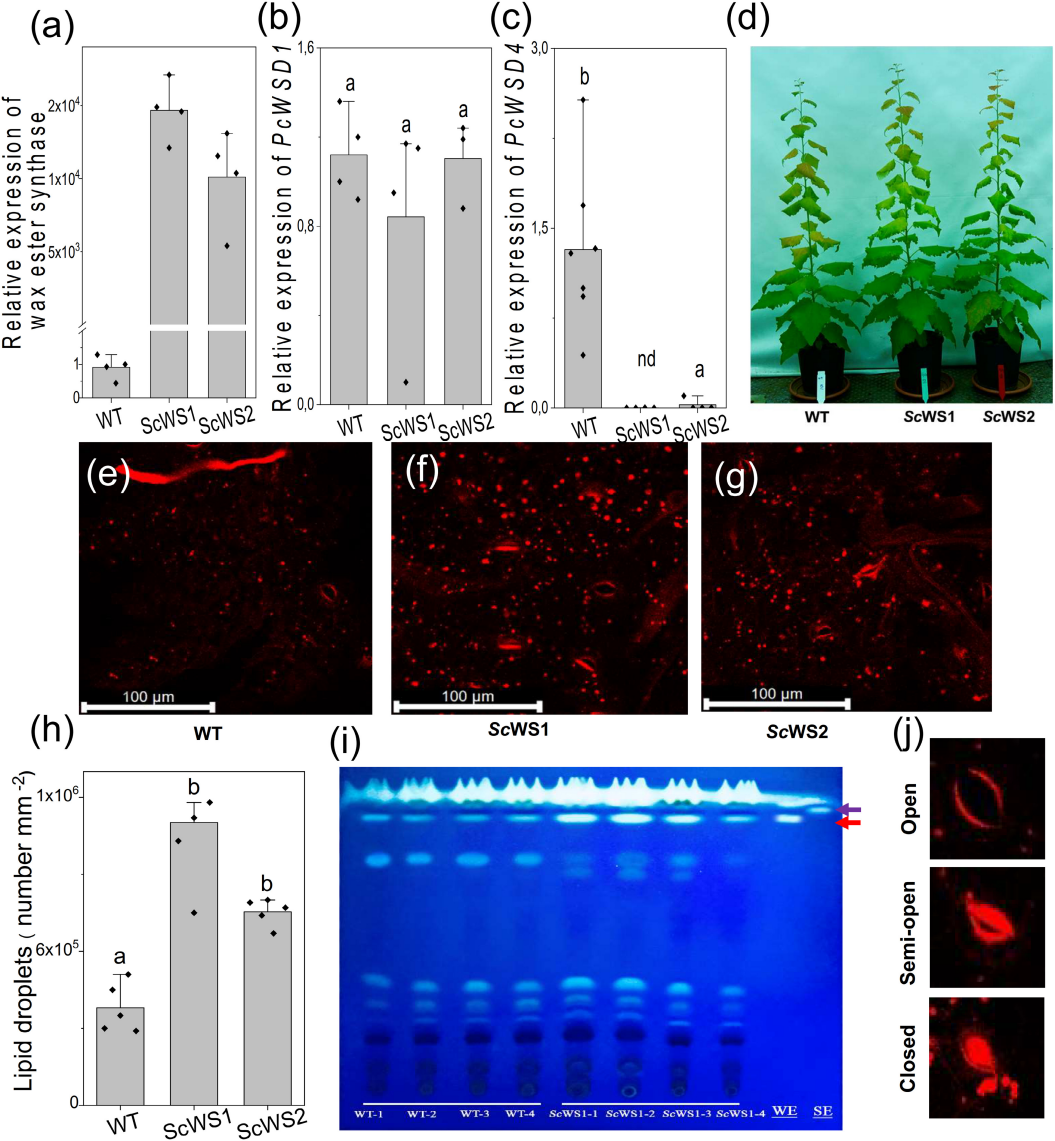
Characterization of jojoba wax ester synthase (*ScWS*) expressing poplar lines compared with the wild type (WT) *P*. × *canescens*. (a) Expression of jojoba wax ester synthase (*ScWS*) under the *35S* promoter in the poplar lines ScWS1 and ScWS2 relative to *PcWSD1* in WT poplars. (b) Expression of *PcWSD1*. (c) Expression of *PcWSD4*; nd = not detected. (d) Phenotypes of WT and *ScWS* expressing lines. (e) Lipid droplets in leaves of WT, (f) in leaves of ScWS1, (g) in leaves of ScWS2 lines. Lipids were visualized by confocal laser scanning microcopy through the leaf after staining with the dye Lipid spot II. (h) Number of lipid droplets per mm^2^ in leaves of WT and *ScWS* lines. Lipid droplets were counted in four areas of 10 × 10 μm^2^ per individual sample and normalized to 1 mm^2^. (i) Thin layer chromatography separation of leaf total lipid extracts from WT and *ScWS* expressing lines. Different lanes indicate samples from individual plants. After separation, the lipids were visualized with primuline under UV light. Wax ester (WE, red arrow) and steryl ester (SE, purple arrow) were used as the standards. (j) Examples for stomatal phenotypes detected in WT and *ScWS* overexpressing lines after lipid staining. Bars show means (± SE) and symbols biological replicates (*n* = 4–6). Different letters indicate significant differences among means at *P* ≤ 0.05 (ANOVA, *post‐hoc* Tukey test).

Poplar (*Populus* × *canescens*) transformed with the jojoba *Sc*WS under the *35S* promoter (called ScWS lines) showed high expression levels of the transgene in several independent lines (Fig. S1). We selected the lines ScWS1 and ScWS2 for our study (Fig. 1a). Since *AtWSD1* is mainly responsible for wax ester production of the cuticle of *A. thaliana* (Li *et al*. 2008), we conducted a phylogenetic analyses of poplar *WSD*s (Fig. S2; conducted according to Cheng *et al*. 2022) to identify close homologues of *AtWSD1*. We chose *PcWSD1* as the reference for wax ester synthesis in WT plants because its expression level was not affected by Sc*WS* expression (Fig. 1b), whereas *PcWSD*4, the closest homologue to *AtWSD1*, showed drastically suppressed transcript levels in the *Sc*WS lines compared with the WT (Fig. 1c).

The growth phenotype of *Sc*WS lines was similar to that of WT plants (Fig. 1d). Sc*WS* was expressed in all inspected poplar tissues (leaf, bark, wood; Fig. S3). The suppression of Pc*WDS4* in the transgenic *Sc*WS lines was specific to leaves and bark, and not found in poplar wood or developing xylem (Fig. S4).

Confocal laser scanning microscopy throughout the whole leaf plane (Fig. S5) after lipid staining revealed denser appearance of lipid droplets in the transgenic lines than in the WT (Fig. 1e–g). ScWS1 contained about 2‐fold and ScWS2 about 1.5‐fold higher numbers of lipid droplets than the WT (Fig. 1h). Thin‐layer chromatography of whole‐leaf lipid extracts support an accumulation of wax esters in ScWS lines compared with WT plants (red arrow, Fig. 1i).

### Sc*WS* ‐expressing poplar lines have partially closed stomata

**Fig. 2:**
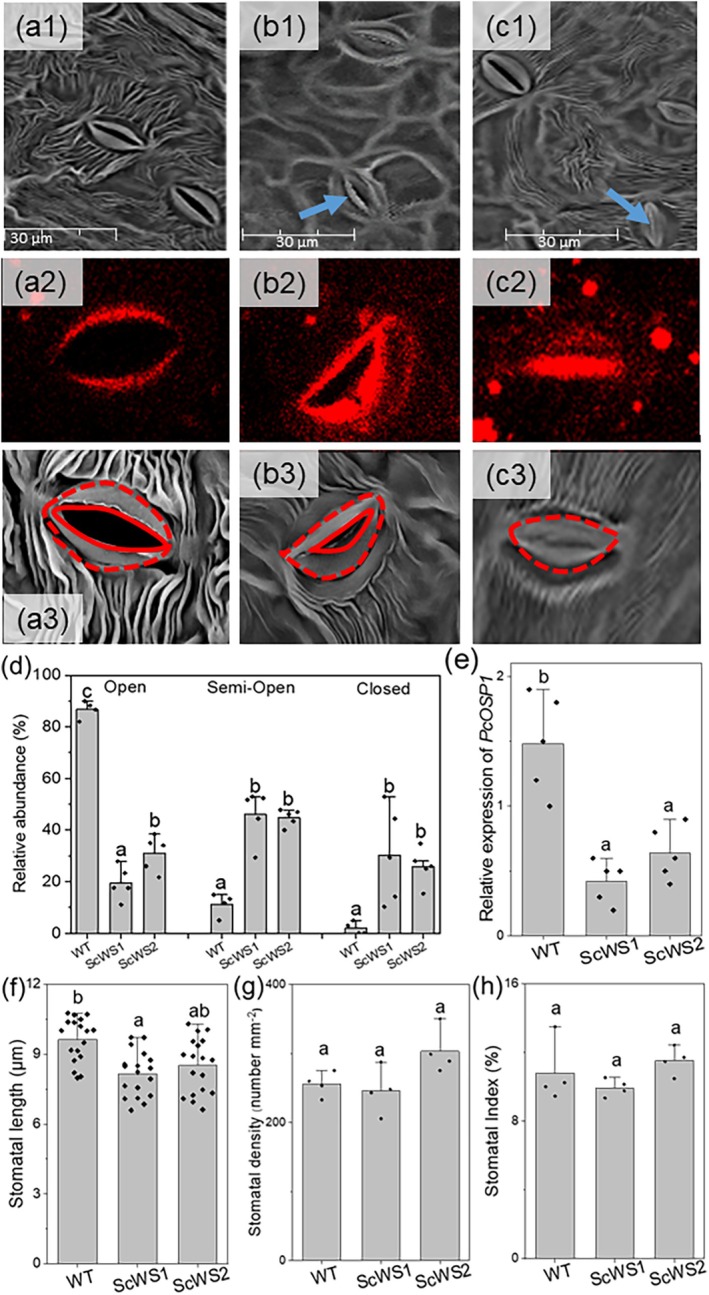
Characterization of stomatal phenotypes in wild type (WT) *P*. × *canescens* and in the jojoba wax ester synthase (*ScWS*) expressing lines. Scanning electron microscopy of the abaxial leaf surface of (a1) WT, (b1) *Sc*WS1 and (c1) *Sc*WS2. Typical views of individual stomata after lipophilic staining of WT (a2) and ScWS lines (b2, c2) and under a scanning electron microscope of WT (a3) and ScWS lines (b3, c3). Note, that some guard cells of *Sc*WS1 and *Sc*WS2 are not correctly opened and the ridges emerging from the guards are less developed than in the WT (blue arrows in b1 and c1). (d) Quantification of stomatal types. The classifications of stomatal types (open, semi‐open, closed) are based on their visual appearance, as shown in a2, b2, and c2. The total number of counted stomata per area unit was set as 100%. (e) Relative expression of *PcOSP1* in leaves of the WT and ScWS overexpressing lines. (f) Stomatal length, (g) stomatal density, (h) stomatal index. Stomatal properties were determined on the abaxial leaf surface. Bars show means (± SE) and symbols are biological replicates (*n* = 4–6). Different letters indicate significant differences among means at *P* ≤ 0.05 (ANOVA, *post‐hoc* Tukey test).

Lipid staining further revealed more intense appearance and irregular patterns at the stomatal pores of guard cells in the transgenic lines compared with the WT (Fig. 1e–g). This phenotype was caused by aberrant lipid accumulation at guard cells (Fig. 1j). SEM and magnification of lipid stains confirmed that stomata of the WT had regular shapes with a clear pore (Fig. 2a). In contrast to the WT, stomata of the ScWS lines often had an irregular shape, suggesting that the guard cells did not separate correctly and therefore were partially blocked (Fig. 2b,c); these stomata accumulated lipophilic material. These phenotypic differences were also found under high humidity for young plants growing under tissue culture conditions (Fig. S6). This result excludes that the decreased stomatal opening of the ScWS lines was caused by increased susceptibility to lower ambient air humidity present under greenhouse conditions. Furthermore, we observed that cuticular ridges extending around the guard cells were less developed in the ScWS lines than in the WT (Fig. 2a–c). Additional overviews on the ad‐ and abaxial leaf surfaces are presented in Fig. S7.

Almost 90% of the WT, but only 20% to 30% of the ScWS, stomata were open (Fig. 2d). In the ScWS lines, about 40% of the stomata were mis‐shaped, resulting in a semi‐open phenotype (Fig. 2d). In the ScWS lines, about 20%–30% of the stomata were closed (Fig. 2d). Furthermore, the ScWS lines had slightly shorter stomata (Fig. 2f), but the stomatal density and the stomatal indices were unaffected compared with the WT (Fig. 2g,h). Since the stomatal features of the ScWS lines resembled those previously observed in an *A. thaliana* null mutant of *OSP1* (*OCCLUDED STOMATAL PORE 1*, Tang *et al*. 2020), we measured the relative transcript abundance of the poplar Pc*OSP1* homologue. We found a strong decrease in the Pc*OSP1* levels of the ScWS lines compared with the WT (Fig. 2e).

We measured ABA concentrations and expression of transcription factors involved in ABA‐mediated wax biosynthesis. The foliar ABA levels and the transcript abundances of Pc*MYB94* were unaffected, and that of Pc*MYB96* even decreased in the *Sc*WS lines compared with the WT (Fig. S8). Increases in ABA are known to result in decreases in the stomatal index (Tanaka *et al*. 2013), but our study showed neither effects of Sc*WS* expression on the ABA concentrations nor on the stomatal index (Fig. 2h).

### Moderate shifts in surface waxes do not affect non‐stomatal water loss of ScWS lines

**Fig. 3:**
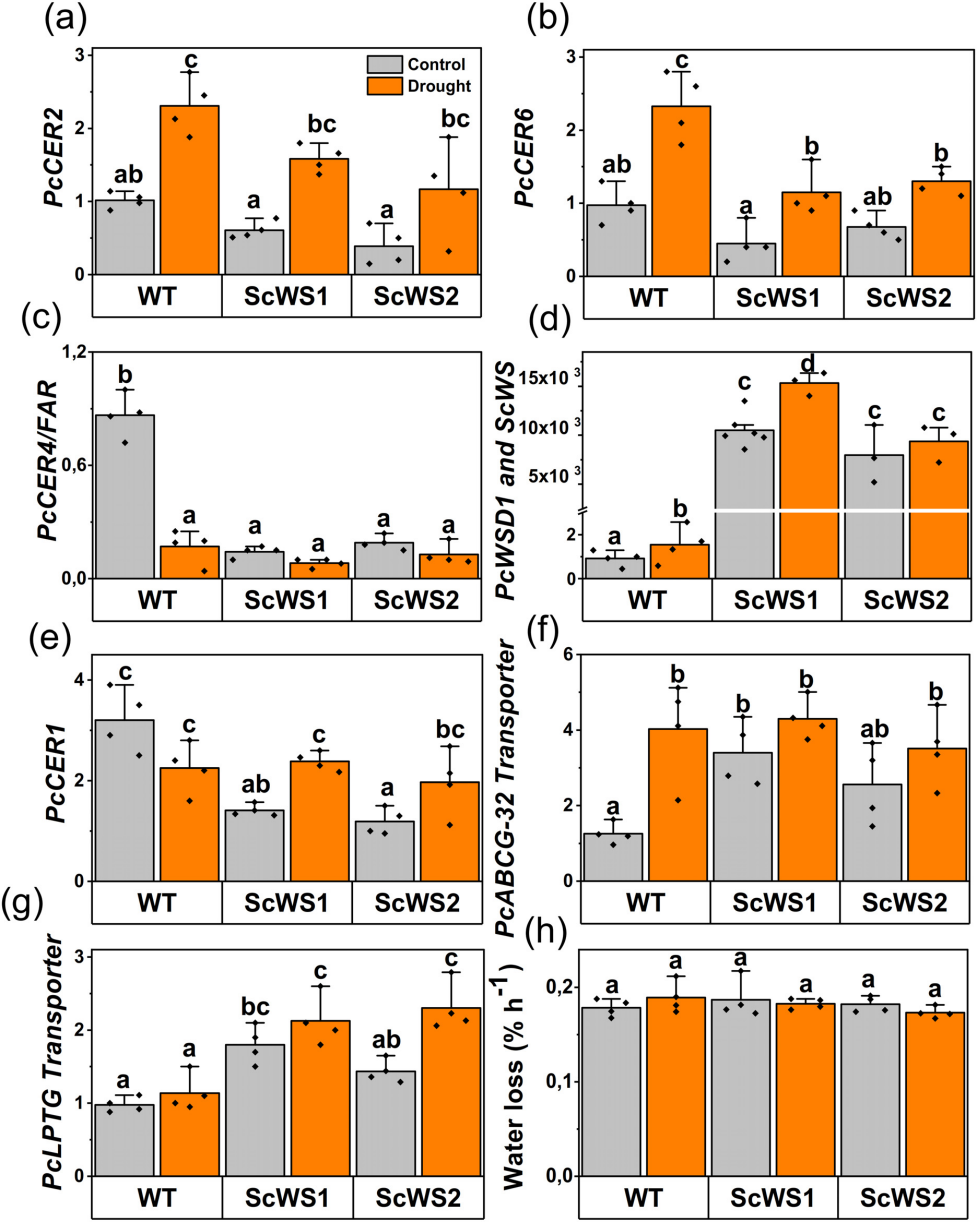
Relative expression levels of genes involved in wax biosynthesis in wild‐type (WT) and *ScWS* expressing poplars under well‐irrigated and drought‐stressed conditions. (a) *PcCER2* and (b) *PcCER6*, which are potentially involved in the elongation of fatty acyl CoA to very long chain (VLC) fatty acyl CoA. (c) *FATTY ACID REDUCTASE* (*PcCER4/FAR3*), which provides the precursors for wax ester synthase, (d) wax ester synthase (*ScWS*) in transgenic lines and *PcWSD1* in the WT, (e) *PcCER1*, which diverts VLCFA into the alkane/alkene pathway, (f) putative lipid transporters of the adenosine triphosphate binding cassette family (*Pc* *ABCG 32*) and (g) of the lipid transfer protein family (*Pc* *LTPG*), (h) velocity of non‐stomatal water loss. Control and stressed leaves were harvested after 21 days of withholding water from the long‐term drought experiment under greenhouse conditions. Bars are means (*n* = 4 individual plants per line and treatment ± SE) and symbols are biological replicates. Data were log‐transformed, analysed by ANOVA and *post‐hoc* Tukey test. Different letters indicate significant differences among the treatments and lines at *P* ≤ 0.05. Grey = well‐irrigated, orange = drought‐stressed.

In the next step, we tested if expression of Sc*WS* affected the biosynthesis and composition of surface waxes under well‐irrigated or drought‐stressed conditions. We studied expression of the biosynthetic key genes involved in the alcohol‐forming pathway leading to wax esters and in the alkane‐forming pathway required for the production of VLC aldehydes, alkanes, and alkenes (Fig. 3). Both pathways start with the elongation of fatty acids (C16/C18 acyl CoA) including, among other enzymes, CER2 and CER6 (Xia *et al*. 1997; Fiebig *et al*. 2000). Under non‐stressed conditions, the transcript levels of *CER2* and *CER6* were not significantly affected in the *Sc*WS lines compared with the WT (Fig. 3a,b). Under drought, however, the transcript levels increased. The increase was generally more pronounced in the WT than in the *Sc*WS lines (Fig. 3a,b).

The resulting VLC acyl‐CoAs are reduced by FAR to primary alcohols and then linked with a fatty acid moiety by WSD for cuticular wax ester production (Rowland *et al*. 2006; Li *et al*. 2008). Here, the transcript levels of Pc*CER4/FAR3* were about nine‐fold lower in the ScWS lines than in the WT under non‐stressed conditions (Fig. 3c). Under drought, the Pc*CER4/FAR3* levels decreased in the WT and remained low in the ScWS lines (Fig. 3c). The Pc*WSD1* transcript levels increased two‐fold in WT leaves in response to drought, but were still more than three orders of magnitude lower than those of the constitutively expressed Sc*WS* in the transgenic lines (Fig. 3d).

After fatty acid elongation, VLC acyl‐CoAs are also converted into VLC aldehydes, alkanes, and alkenes. The production of alkanes is promoted by CER1, synergistically with other enzymes (Aarts *et al*. 1995; Bourdenx *et al*. 2011). In WT poplars, Pc*CER1* transcript levels were not significantly affected by drought (Fig. 3e). In the non‐stressed ScWS lines, the transcript levels of Pc*CER1* were about two‐fold lower than in the WT but increased to WT levels in response to drought (Fig. 3e).

The transcript abundances of transporters for lipophilic compounds to the leaf surface (Pc*ABCG32, PcLTPG*) increased Sc*WS* lines and under drought in comparison with the WT (Fig. 3f,g).

The water loss assay, which indicates the velocity of non‐stomatal water loss, did not reveal significant differences compared with WT leaves (Fig. 3h).

**Fig. 4:**
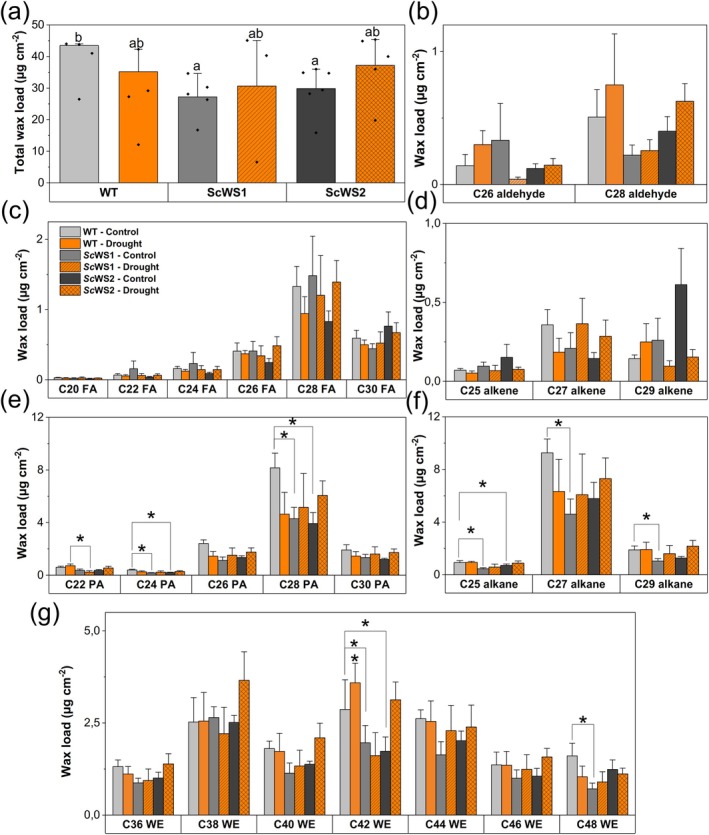
Total wax load and composition of cuticle waxes of *P*. × *canescens* leaves of the wild type (WT) and the transgenic lines *Sc*WS1 and *Sc*WS2. (a) total wax load. (b) aldehydes, (c) fatty acids (FA), (d) alkenes, (e) primary alcohols (PA), (f) alkanes, and (g) wax esters, with Cn showing aliphatic carbon chain lengths. Different letters above bars in panel (a) indicate significant differences of means at *P* ≤ 0.05 (ANOVA, *post‐hoc* least significant difference test). Panels b‐g: Bars show means (± SE) with *n* = 5 biological replicates per line and treatment (exceptions: WT control *n* = 4, *Sc*WS2 drought *n* = 3) and stars indicate significant differences between treatment and control for the individual compounds (*post‐hoc* Student's *t*‐test, *P* ≤ 0.05). Control and stressed leaves were harvested after 21 days of withholding water from the long‐term drought experiment under greenhouse conditions. Grey bars = well‐irrigated, orange bars = drought‐stressed. Light colour = WT, hatched = *Sc*WS1, rhombic‐hatched = *Sc*WS2.

We investigated the influence of Sc*WS* expression on cuticular wax composition and total wax load under well‐irrigated and drought‐stressed conditions (Fig. 4). In the WT, the main categories were wax esters (13.4 ± 1.6 μg cm^−2^), primary alcohols (9.9 ± 1.5 μg cm^−2^), and alkanes (8.9 ± 1.9 μg cm^−2^). Overall, the total cuticular wax load was lower in ScWS lines than in the WT (Fig. 4a). The reductions were mainly caused by decreases in alkanes (C25, C27, and C29), primary alcohols (C22, C24, C28), and wax esters (C42, C48) in the ScWS lines, compared with the WT (Fig. 4e–g). At the level of individual compounds, these reductions were often only significant in the line ScWS1 (Fig. 4e–g). Other constituents of the cuticular waxes (fatty acids, aldehydes, alkenes) had low abundances and did not show significant differences among lines (Fig. 4b–d). Leaf cross‐sections inspected under TEM did not reveal changes in thickness of the cuticular wax layer (Fig. S9).

Drought caused marginal decreases in the surface waxes in the WT and marginal increases in ScWS lines (Fig. 4). Consequently, the differences in the total wax load among the WT and the ScWS lines disappeared under drought (Fig. 4a).

### Sc*WS* ‐expressing poplars exhibit enhanced water‐use efficiency but long‐term growth penalty

**Fig. 5:**
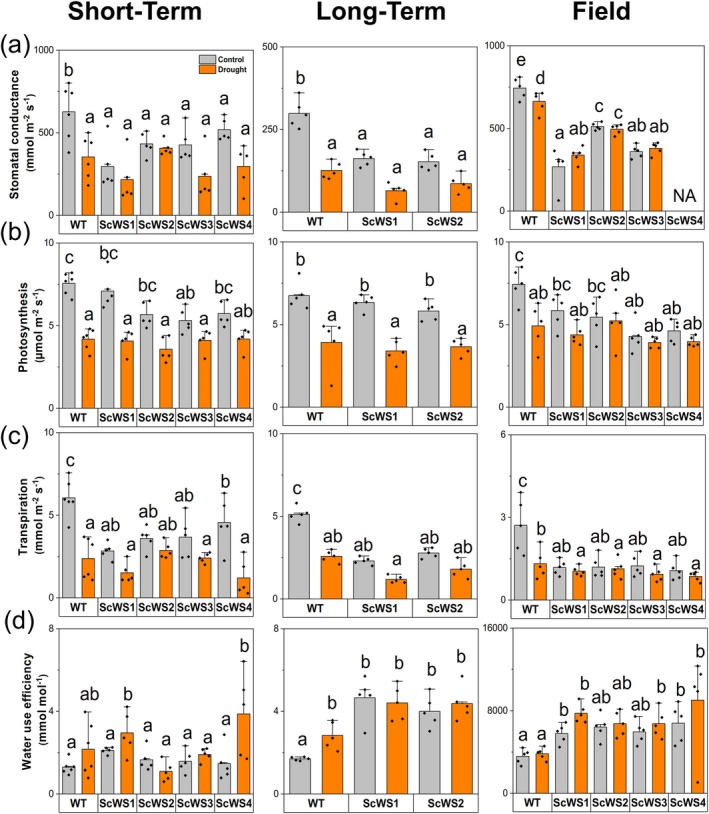
Stomatal conductance (a), photosynthesis (b), transpiration (c) and water‐use efficiency (WUE) (d) of *P*. × *canescens* (WT) and *ScWS* expressing lines in short‐term, long‐term and outdoor drought experiments. Gas change measurements were conducted once per week and stomatal conductance every second day from 10:00 to 15:00 h during the drought phase. NA = not available. Grey bars indicate control and orange bars drought‐stressed conditions of the WT and the *ScWS* lines. Bars indicate means (± SE) and symbols the biological replicates (*n* = 5 per line and per treatment). Different letters indicate significant differences at *P* ≤ 0.05 (ANOVA, *post‐hoc* Tukey test).

Under well‐irrigated conditions (soil moisture >0.3 m^3^ m^−3^), stomatal conductance of WT plants ranged from 600 to 800 mmol m^−2^ s^−1^ and was almost two times higher than that of the ScWS lines (Fig. 5a). Under drought (soil moistures of ca. 0.1 m^3^ m^−3^), stomatal conductance of the WT lines declined to levels similar to those of the non‐stressed transgenic lines (Fig. 5a). These response patterns were found in different independent experiments by withholding water for increasing drought periods from 1 week (short‐term greenhouse) to 3 weeks (long‐term greenhouse) or almost 2 months under field conditions in a plantation‐like growth array (Fig. 5a, Fig. S10).

Although stomatal conductance in the ScWS lines was about two‐fold lower than in the WT under well‐irrigated conditions, photosynthesis was not significantly affected (mean decrease: −15%; Fig. 5b). Under drought, photosynthesis decreased more strongly in the WT than in ScWS lines, reaching similar levels across WT and transgenic poplars (Fig. 5b). To gain deeper insight into the photosynthetic behaviour of ScWS lines, we measured CO_2_ assimilation under increasing light intensities. We found that the ScWS lines and the WT exhibited similar photosynthesis rates under low light intensities, but under higher light intensities (≥200 μmol m^−2^ s^−1^ PAR), the WT had higher photosynthesis than the ScWS lines (Fig. S12). This finding indicates that light‐limited photosynthesis was not additionally lessened by the reduced stomatal conductance of the ScWS lines. Nighttime stomatal conductance and transpiration were low and did not differ between the ScWS lines and the WT, neither under greenhouse nor under field conditions (Tables S2 and S3). Thus, the regulation of stomatal closure was not hindered in the *Sc*WS lines.

In accordance with the highest stomatal conductance, well‐irrigated WT plants also had the highest transpiration rates (Fig. 5c) and lowest instantaneous water‐use efficiencies (Fig. 5d). Under drought, WT plants showed either no or small increases (+20%) in instantaneous water‐use efficiencies (Fig. 5d). With the exception of short‐term experiments, ScWS poplars exhibited higher instantaneous water‐use efficiencies than the WT poplars (Fig. 5d). The largest instantaneous water‐use efficiencies were observed for ScWS poplars during drought stress under outdoor conditions (Fig. 5d).

**Fig. 6:**
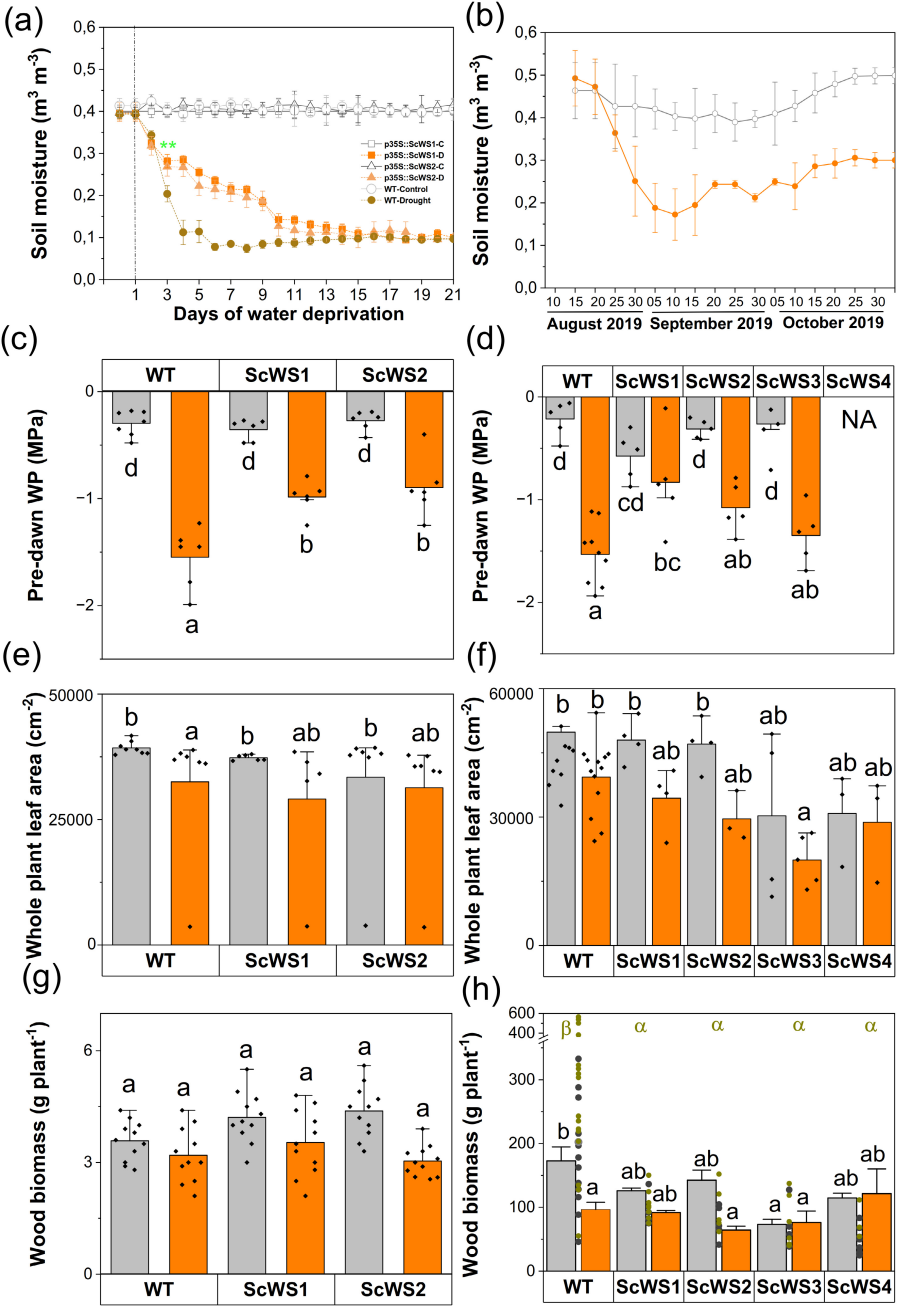
Soil moisture (a, b), pre‐dawn water potential (c, d), whole‐plant leaf area (e, f) and wood biomass (g, h) of *P*. × *canescens* wild type (WT) and *ScWS* expressing lines in a long‐term greenhouse and an outdoor drought experiment. Soil moisture was measured regularly. The predawn water potential was measured at night, before dawn, using plants 12 days (greenhouse experiment) and 8 weeks after withholding water (outdoor experiment). Data show means (± SE) and black symbols the biological replicates (*n* = 5 per line and per treatment). Whole‐plant leaf area and wood biomass (stem plus branches) were determined at the time point of harvest (means ± SE of *n* = 6 individuals in the greenhouse experiment and n = 5 to 10 in the outdoor experiment 2019) per line and treatment. Grey bars = well‐irrigated, orange bars = drought stress. Different letters above the bars indicate significant differences at *P* ≤ 0.05 (ANOVA of log‐transformed data, *post‐hoc* Tukey test). In panel (h), bars indicate mean biomass in the first year (2019) and symbols biomass of individual plants in the second year (2020). In 2020, all plants were well irrigated (dark grey symbols = well irrigated in 2019, dark yellow symbols = drought‐stressed in 2019). Transformation did not result in homogeneity of variances. Therefore, the data were analysed by the non‐parametric Kruskal Wallis test. Different greek letters in (h) indicate differences at *P* < 0.05 in 2020.

We further characterized the impact of Sc*WS* expression on water consumption. We determined soil moisture, predawn water potentials, and whole‐plant leaf area as decisive factors for plant water balance. When the plants were subjected to long‐term drought under greenhouse conditions, the WT plants showed a faster decrease in soil moisture than the ScWS lines (Fig. 6a). This indicates higher water consumption per WT plant. Higher water‐spending of the WT poplars further resulted in a stronger decline in the predawn water potential than in the ScWS poplars (Fig. 6b). Since whole‐plant leaf areas (Fig. 6c), plant stem heights (mean height WT: 95.5 ± 1.4 cm and *Sc*WS lines: 87.7 ± 1.9 cm), and non‐stomatal water loss (Fig. 3f) did not differ between WT and *Sc*WS lines, the water‐saving phenotype of the ScWS lines can be attributed to lower stomatal conductance. After 3 weeks of drought under greenhouse conditions, the WT poplars had about 25% less leaf area than well‐watered WT plants, whereas the reductions were not significant for the transgenic lines (Fig. 6e). WT leaf biomass was 20% decreased under drought (7.8 ± 0.2 g) compared to that of the ScWS lines (9.7 ± 0.3, *P* = 0.035; Kruskal Wallis test), mainly because of increased leaf shedding, whereas stem biomass was unaffected (Fig. 6g).

In contrast to plants in individual pots, the outdoor plants were grown in mixtures of WT and ScWS lines, sharing large soil volumes (Fig. S10). The water consumption of individual plants could therefore not be assessed. Withholding water resulted in slow changes in soil moisture (Fig. 6e) and caused similar reductions in the pre‐dawn water potentials of leaves from WT and ScWS lines (Fig. 6f). ScWS and WT had similar leaf areas, which were not significantly affected by drought (Fig. 6g).

In long‐term field studies, stable transgene expression is a matter of concern (Li *et al*. 2009). Here, Sc*WS* expression of the field‐grown plants was three orders of magnitude higher than that of *PcWDS1* in the WT (Fig. S11).

At the end of the field season, the aboveground plant biomass was harvested. Since the position of the plants (inside or at the edge of the plantation) did not affect biomass production (*P* = 0.294, Kruskal Wallis test), all plants were included in the analyses. Well‐irrigated WT produced the largest stem biomass and line ScWS3 the least, while the ScWS lines 1, 2, and 4 had an intermediate stem biomass (bars in Fig. 6h). Across all treatments, drought caused a decline in wood biomass (*P* = 0.0003, Kruskal Wallis test). However, for a given genotype, only the WT biomass was significantly reduced, while none of the ScWS lines showed significant drought‐induced reductions (Fig. 6h).

In short‐rotation plantation practice, the ability of stumps to produce new shoots after aboveground harvest is an important feature. In the following growth season, we observed the re‐sprouting of new shoots in 87% of the ScWS lines and in all WT plants. During the growth season, gas exchange of the ScWS lines and the WT was similar to that in the previous season; however, with a stronger reduction of photosynthesis (Table S4). At the end of the growth season, the woody biomass of some ScWS lines was up to 65% lower than that of the WT (individual data points in Fig. 6h). Previously drought‐stressed plants did not show significantly different woody biomass compared with well‐irrigated plants (*P* = 0.061, Kruskal Wallis test).

## DISCUSSION

### ScWS lines exhibit increased intracellular wax ester accumulation but decreased cuticular wax loads

Wax esters are particularly valuable commodities used for multiple specialized industrial purposes. In this study, we show that *P*. × *canescens*, an important non‐food biomass crop, accumulates lipids in green tissues after transformation with jojoba wax ester synthase (*Sc*WS). This is an important result, confirming that the *Sc*WS enzyme is active *in planta*. This finding supports that vegetative tissues may serve as a platform for biotechnological production of lipids (Vanhercke *et al*. 2019). Previous investigations focused on approaches to increase wax ester concentrations in seeds using model plants (*A. thaliana*) and crop plants such as *Brassica carinata*, *Lepidium campestre, Crambe abyssinica*, and *C. sativa* (Iven *et al*. 2016; Zhu *et al*. 2016; Ivarson *et al*. 2017; Yu *et al*. 2018; Tesfaye *et al*. 2024).

A significant enrichment of wax esters in *A. thaliana* and *C. sativa* required co‐transformation with FAR enzymes, suggesting that the production of wax esters in seeds was limited by the availability of precursors (Iven *et al*. 2016; Vollheyde *et al*. 2021). Enhanced production of wax esters in leaves of *Nicotiana benthamiana* also required *FAR* coexpression (Domergue & Miklaszewska 2022). Our study shows that in poplar *FAR/*Sc*WS* coexpression was not necessary to enhance the intracellular lipids, since the leaves of the ScWS lines contained increased lipid droplets. The accumulation of lipid droplets may reflect the enhanced wax ester levels detected by chromatography or could be the result of increased triacylglycerol production, which can occur via bifunctional activities of some wax ester synthases (Kalscheuer & Steinbuchel 2003; Barney *et al*. 2012). In addition to strongly decreased expression levels of Pc*CER4/FAR3* compared with the WT (cf. Fig. 7), the Sc*WS* expression caused downregulation of Pc*WSD4* and Pc*CER1*. The transcriptional alterations of the cuticular wax biosynthetic enzymes were associated with decreased amounts of distinct compounds in the cuticle, i.e., primary alcohols (C22, C24, C28), alkanes (C25, C27), and wax esters (C42, C48). Since wax components of alcohol‐ and alkane‐forming pathways were reduced, we speculate that the intracellular accumulation of wax esters may cause feedback inhibition of cuticular wax biosynthesis, possibly already close to the entrance of the pathway. It is possible that coordinated downregulation of biosynthesis enzymes co‐limits the two branches of wax biosynthesis (Yang *et al*. 2022). The decrease in alkanes is suspicious because they are main constituents of poplar cuticular waxes (Grünhofer *et al*. 2022, our study). However, the reductions in alkanes were too small to affect permeability of the cuticle. Whether the reductions in wax load in the ScWS lines were the result of reduced activities of *PcFAR, PcCER1*, and *PcWSD4* requires further investigations.

Another possibility, which may restrict the export of lipophilic compounds to the external leaf surface, are limiting capacities of transporters. However, we found that transcript levels of homologues to *AtABCG11* (*PcABCG32*) and lipid transfer protein GPI anchored 12 (*PcLTPG*) (Pighin *et al*. 2004; DeBono *et al*. 2009) were increased in the ScWS‐expressing lines compared with the WT. This result does not support restrictions on the export of aliphatic compounds. Although transcript levels may not reflect actual transport activities, we would not expect reductions in wax load, as observed here.

Previous studies report that not only the amounts of cuticular waxes but also their composition are decisive to prevent uncontrolled non‐stomatal water loss (Schreiber & Riederer 1996; Riederer & Schreiber 2001; Meng *et al*. 2019; Seufert *et al*. 2022; Grünhofer *et al*. 2024). For example, Grünhofer *et al*. (2024) studied the surface waxes of *P*. × *canescens* in a CRISPR/Cas9 *cer6* mutant. The *cer6* mutants showed massively decreased levels >C24 wax monomers, increased >C34 wax ester dimers, and enhanced non‐stomatal water loss (Grünhofer *et al*. 2024). These authors speculated that the shifts in wax composition might result in structural disturbance to the surface that allow non‐specific water loss. In our study, *CER6* expression was only marginally reduced in the ScWS lines, and increased in the WT and the transgenic lines under drought; however, without any effects on the amount of C44 wax esters. Drought further induced increased levels of Pc*CER2* and Pc*WSD1* and obliterated differences in composition of the WT and ScWS cuticles. As non‐stomatal water loss was not affected, our findings support that poplars regulate wax biosynthesis to maintain functional homeostasis under drought stress. Obviously, expression of Sc*WS* in poplar was not able to reinforce the cuticle to an extent achieved, for example, by overexpression of At*WDS1* in *A. thaliana* and *C. sativa* (Abdullah *et al*. 2021). In conclusion, our initial speculation that overexpression of Sc*WS* may push wax biosynthesis towards enhanced cuticular wax loads was not supported. Instead, we found an accumulation of intracellular lipids, which hold promise for biotechnological applications. This potential of our novel *Sc*WS poplar lines deserves further attention but is beyond the scope of the present study.

### ScWS lines exhibit an occluded stomatal phenotype and show improved water‐use efficiency

An important unexpected result of our study was that *ScWS* expression in poplar led to partially closed stomata. ABA is a key hormone controlling stomatal opening (Cotelle & Leonhardt 2019). ABA can affect wax biosynthesis via regulation of the transcription factors MYB94 and MYB96 (cf. scheme in Fig. S8; Seo *et al*. 2011; Lewandowska *et al*. 2024). Here, we had no evidence that these pathways were affected in the ScWS lines. This speaks against involvement of ABA regulation in the stomatal phenotype of the ScWS lines.

We noted an accumulation of lipids on the guard cells, phenocopying the “occluded stomata syndrome” (Gray *et al*. 2000; Hunt *et al*. 2017; Tang *et al*. 2020). Our histochemical and electron microscopy analyses show that the stomatal pores were not formed properly, and that the affected guard cells exhibited massive lipid enrichment. During normal development, cuticle waxes extend at the upper side of the ventral wall around the pore, forming so‐called outer cuticle ledges (Hunt *et al*. 2017). The formation of the ledges is controlled by FOCL1 (FUSED OUTER CUTICLE LEDGES 1) (Hunt *et al*. 2017) and OSP1 (Tang *et al*. 2020). Null mutants of these genes independently cause a large fraction of occluded stomata. Their phenotype strongly resembles that found here in the *ScWS*‐expressing poplars. In our study, *ScWS* expression caused drastic suppression of *PcOSP1*, suggesting a lack of OSP1 might have been the cause of the observed stomatal syndrome. Whether the *ScWS* under the constitutive *S35* promoter show different local activities in epidermal and guard cells with the observed downstream consequences for *OSP1* expression and stomatal development, remains to be elucidated. Tang *et al*. (2020) proposed that OSP1 (a GDSL lipase) may be an acyl‐CoA thioesterase since it can hydrolyze C22:0 and C26:0 acyl‐CoAs *in vitro* (Tang *et al*. 2020). At*OSP1* was localized to the guard cells and appeared to be part of the alkane forming pathway, acting upstream of CER1 (Tang *et al*. 2020). The exact molecular mechanisms *in planta* that result in stomatal occlusion are still unknown but point in concert with our study and other investigations (Gray *et al*. 2000; Hunt *et al*. 2017; Tang *et al*. 2020) to a central role of wax biosynthesis as a hub controlling cuticle enforcement jointly with correct stomatal development.

The *A. thaliana osp*1 mutant has a reduced cuticular wax load; it exhibits increased non‐stomatal evaporation but decreased stomatal transpiration (Tang *et al*. 2020). In our study, we also found a reduced wax load but non‐stomatal water loss from leaves and night respiration did not differ among the transgenic ScWS lines and the WT, supporting that the moderate decrease in total cuticular wax load observed in the transgenic poplar ScWS lines did not influence the cuticular barrier properties for water. Our study suggests that the reduced stomatal conductance of the ScWS lines was caused by incomplete pore formation and accumulation of lipids. These alterations might have contributed to partial closure of stomata in the *ScWS* lines.

Poplars are high water‐spending species (Xi *et al*. 2021). Here, the ScWS lines show a significant increase in water‐use efficiency, which results in decreased whole‐plant water consumption. Thereby, the time is extended until the plants suffer from drought stress, as evident in leaf shedding. Since no significant influence on the non‐stomatal water loss was found, and the stomatal densities and stomatal indices were unaffected, the improved water economy of the *Sc*WS lines was caused by partially closed stomata. In accordance with transgenic *A. thaliana* plants with occluded stomata (Hunt *et al*. 2017; Tang *et al*. 2020), the almost two‐fold reduction in stomatal conductance of the transgenic *Sc*WS poplar lines initially had only little or no negative effects on photosynthesis or growth. The apparent absence of growth reductions was surprising. However, light curves of photosynthesis showed that carbon assimilation was not affected by stomatal limitations at moderate light intensities. Moderate light is typical for cloudy conditions. Photosynthesis was only reduced at light saturation. This finding implies that optimum photosynthetic conditions cannot be fully exploited. Under variable weather conditions in the field, this disadvantage is temporally limited in the *Sc*WS lines, whereas the benefit of less water consumption persists. In our study, growth reductions occurred in the second growth cycle, most likely as the cumulative result of moderate photosynthetic reductions of *Sc*WS lines over time. Yet, the improved water‐use efficiency is expected to be of a huge benefit for plantations, which are currently irrigated using tap‐ on ground‐water, creating problems for sustainable crop management (Wilske *et al*. 2009; Folch & Ferrer 2015; Xi *et al*. 2021).

In conclusion, our study provides evidence that bioengineering of wax biosynthesis in poplar opens a promising avenue for biotechnological applications in vegetative tissue of plantation trees. This has the potential to improve water management of plantations in a changing, drier climate. However, towards this goal, a deeper understanding of the molecular mechanisms is required to enable fine‐tuning of the dedicated genes. To date, only three independent studies (Hunt *et al*. 2017; Tang *et al*. 2020; our study), each targeted at a different enzyme, show that increased water‐use efficiency can be achieved through increased wax accumulation by the guard cells. Our study provides additional insights, revealing that poplars with this syndrome have stable ScWS expression, reasonable fitness, but reduced productivity under field conditions. An obvious next step is better control of stomatal wax accumulation, for example, under a drought‐inducible promoter to optimize the balance between water‐use efficiency and growth.

## AUTHOR CONTRIBUTIONS

AA: conducted experiments, analysed molecular and physiological data, synthesized data, wrote the first draft. GJS: generated transgenic poplars, conducted experiments, analysed data. AK and CH: measured and analysed waxes and phytohormones. FH: measured and analysed SEM data. UL: measured and analysed TEM data. IF: supervision, fund acquisition, scientific advice. AP: research design, supervision, fund acquisition, scientific advice, manuscript writing. All authors commented on and approved the final version of this manuscript.

## FUNDING INFORMATION

We acknowledge funding provided by the IRTG ProTect, GRK2172 (project B2.1, M2.2, Deutsche Forschungsgemeinschaft), the Lichtenberg Research program (Niedersächsisches VW Vorab), and publication funds of the University Göttingen supported by DFG grants to project DEAL.

## CONFLICT OF INTEREST

The authors declare no conflict of interest.

## Supporting information


**Fig. S1.** Relative expression levels of *ScWS* under the *35S* promoter in several transgenic *P*. × *canescens* lines.
**Fig. S2.** Phylogenetic analysis of bifunctional wax synthase/diacylglycerol acyl transferases (WSD) in *P. trichocarpa* and *A. thaliana*.
**Fig. S3.** Expression of Pc*WSD1* in the WT and of Sc*WS* in the transgenic lines in leaves, wood, bark, and developing xylem.
**Fig. S4.** Expression of *PcWSD1* and *PcWSD4* in different tissues of *P*. × *canescens*.
**Fig. S5.** Confocal laser scanning fluorescence microscopy throughout the plane of leaves of *P*. × *canescens* WT and *Sc*WS lines after lipid staining.
**Fig. S6.** Morphology of stomata of WT and transgenic *P*. × *canescens* lines under high humidity of sterile plantlets from tissue culture.
**Fig. S7.** Scanning electron microscopy of the ad‐ and abaxial leaf surface of the WT and ScWS lines of *P*. × *canescens*.
**Fig. S8.** Scheme for the abscisic acid (ABA) signalling pathway, relative expression of *MYB96* and *MYB94* and concentrations of ABA in *ScWS* lines and WT *P*. × *canescens*.
**Fig. S9.** Transmission electron microscopy of cross‐sections of *P*. × *canescens* leaves of the WT and *ScWS* lines.
**Fig. S10.** Planting scheme of WT and *ScWS* lines of *Populus* × *canescens* in mixtures under outdoor conditions.
**Fig. S11.** Relative expression levels of *ScWS* in transgenic poplars and of Pc*WSD1* in WT poplars under outdoor conditions.
**Fig. S12.** Light response curve of photosynthesis of ScWS lines and WT *P*. × *canescens*.
**Table S1.** List of primers and Potri numbers for genes used for the cloning and for expression analyses by qRT PCR.
**Table S2.** Nighttime respiration, transpiration, and stomatal conductance of well‐irrigated and drought‐stressed *ScWS* lines and WT *P. × canescens* in a long‐term greenhouse experiment.
**Table S3.** Nighttime respiration, transpiration, and stomatal conductance in darkness of well‐irrigated and drought‐stressed *ScWS* lines and WT *P*. × *canescens* under field conditions.
**Table S4.** Gas exchange of *P. × canescens* WT and *ScWS* lines under outdoor conditions in 2020 (second growth phase).
**Supporting Methods S1.** Protocols for poplar transformation, scanning electron microscopy, and for cuticular wax analysis.

## Data Availability

Data have been deposited in Figshare DOI: 10.6084/m9.figshare.25549702. ScWS poplar lines are available upon reasonable request from A. Amirkhosravi (Forest Botany and Tree Physiology, University of Göttingen). Accession numbers: Potri.014G152300, Potri.001G319200, Potri.004G185000, Potri.008G120300, Potri.001G304600, Potri.014G098900, Potri.014G160100, Potri.004G126700, Potri.017G082500, Potri.001G311300, Potri.009G158100, Potri.012G141400, Potri.015G001600.
