# Supplementary material for: Overexpression of jojoba wax ester synthase in poplar increases foliar lipid accumulation, alters stomatal conductance, and increases water use efficiency
Source: Plant Biol (Stuttg). 2025 May 27;27(6):1006–21. doi: 10.1111/plb.70056 (PMC12477317; doi:10.1111/plb.70056)
Supplement: Supplementary file 1 — Fig. S1. Relative expression levels of ScWS under the 35S promoter in several transgenic P. × canescens lines. Fig. S2. Phylogenetic analysis of bifunctional wax synthase/diacylglycerol acyl transferases (WSD) in P. trichocarpa and A. thaliana. Fig. S3. Expression of PcWSD1 in the WT and of ScWS in the transgenic lines in leaves, wood, bark, and developing xylem. Fig. S4. Expression of PcWSD1 and PcWSD4 in different tissues of P. × canescens. Fig. S5. Confocal laser scanning fluorescence microscopy throughout the plane of leaves of P. × canescens WT and ScWS lines after lipid staining. Fig. S6. Morphology of stomata of WT and transgenic P. × canescens lines under high humidity of sterile plantlets from tissue culture. Fig. S7. Scanning electron microscopy of the ad‐ and abaxial leaf surface of the WT and ScWS lines of P. × canescens. Fig. S8. Scheme for the abscisic acid (ABA) signalling pathway, relative expression of MYB96 and MYB94 and concentrations of ABA in ScWS lines and WT P. × canescens. Fig. S9. Transmission electron microscopy of cross‐sections of P. × canescens leaves of the WT and ScWS lines. Fig. S10. Planting scheme of WT and ScWS lines of Populus × canescens in mixtures under outdoor conditions. Fig. S11. Relative expression levels of ScWS in transgenic poplars and of PcWSD1 in WT poplars under outdoor conditions. Fig. S12. Light response curve of photosynthesis of ScWS lines and WT P. × canescens. Table S1. List of primers and Potri numbers for genes used for the cloning and for expression analyses by qRT PCR. Table S2. Nighttime respiration, transpiration, and stomatal conductance of well‐irrigated and drought‐stressed ScWS lines and WT P. × canescens in a long‐term greenhouse experiment. Table S3. Nighttime respiration, transpiration, and stomatal conductance in darkness of well‐irrigated and drought‐stressed ScWS lines and WT P. × canescens under field conditions. Table S4. Gas exchange of P. × canescens WT and ScWS lines under outdoor conditions [file PLB-27-1006-s001.docx]

**Supporting Materials**

**Overexpression of jojoba wax ester synthase in poplar increases foliar lipid accumulation, alters stomatal conductance and increases water use efficiency**

Ashkan Amirkhosravi, Gerrit-Jan Strijkstra, Alisa Keyl, Felix Häffner, Ulrike Lipka, Cornelia Herrfurth, Ivo Feussner, Andrea Polle^*^

**Supporting Figures**

**Figure S1.** Relative expression levels of *ScWS* under the *35S* promoter in several transgenic *P*. x *canescens* lines.

**Figure S2.** Phylogenetic analysis of bifunctional wax synthase/diacylglycerol acyl transferases (WSD) in *P. trichocarpa* and *Arabidopsis thaliana.*

**Figure S3.** Expression of Pc*WSD1* in the wild type and of Sc*WS* in the transgenic lines in leaves, wood, bark and developing xylem.

**Figure S4.** Expression of *PcWSD1* and *PcWSD4* in different tissues of *P*. x *canescens.*

**Figure S5.** Confocal laser scanning fluorescence microscopy throughout the plane of leaves of *P*. x *canescens* wild type and *Sc*WS lines after lipid staining.

**Figure S6.** Morphology of stomata of wild type and transgenic *P*. x *canescens* lines under high humidity. **Figure S7.** Scanning electron microscopy of the ad- and abaxial leaf surfaces of the wild type and ScWS lines of *P*. x *canescens*.

**Figure S8.** Scheme for the abscisic acid (ABA) signaling pathway, relative expression of *MYB96* and *MYB94* and concentrations of ABA in ScWS lines and wild type *P.* x *canescens*.

**Figure S9.** Transmission electron microscopy of cross sections of *P*. x *canescens* leaves from the wild type and the ScWS lines.

**Figure S10.** Planting scheme of wild type and ScWS lines of *Populus* × *canescens* in mixtures under outdoor conditions.

**Figure S11.** Relative expression levels of *ScWS* in transgenic poplars and of *PcWSD1* in wild type poplars under outdoor conditions.

**Figure S12.** Light response curve of photosynthesis of *ScWS* lines and wild type *P*. x *canescens*.

**Supporting tables.**

**Table S1:** Night respiration, transpiration and stomatal conductance in darkness of well-irrigated and drought-stressed ScWS lines and wild type *P*. x *canescens* under field conditions.

**Table S2**. Gas exchange of *P. x canescens* wild type and ScWS lines under outdoor conditions in the second growth year.

**Table S3.** List of the primers and Potri numbers for genes used for the cloning and for expression analyses by qRT PCR.

**Table S4.** Night respiration, transpiration and stomatal conductance of well-irrigated and drought-stressed ScWS lines and wild type P. x canescens in a long-term greenhouse experiment.

**Supporting Experimental Procedures.**

**Supporting Methods S1:** Protocols for poplar transformation, scanning electron microscopy of fresh leaf surfaces and for cuticular wax analysis.





**Supplementary Figure S1. Relative expression levels of *ScWS* under the *35S* promoter in several transgenic *P*. *x canescens* lines**. Individual leaves from young plantlets from different transformation events were used.


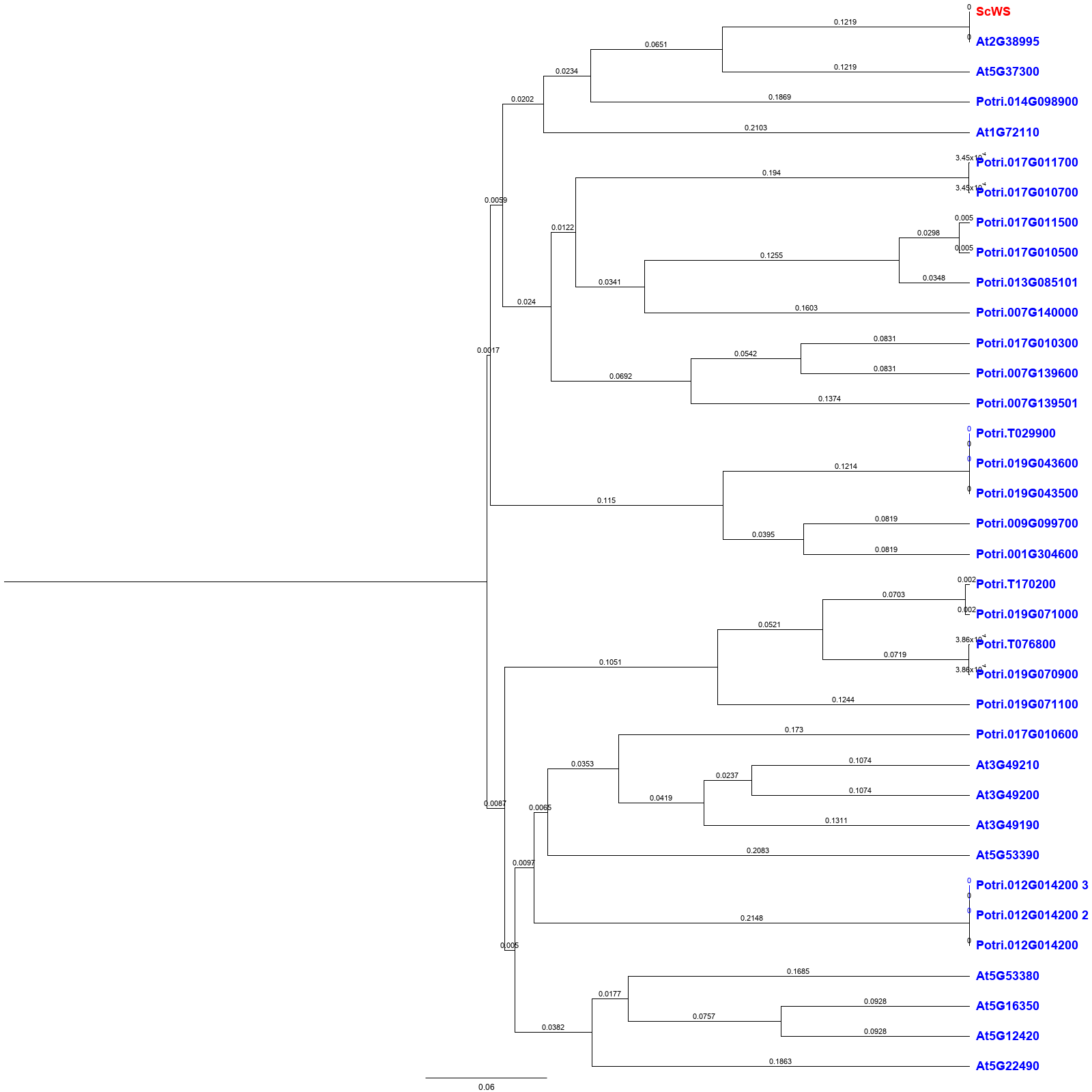


*At*WSD1

*Pc*WSD4

*Pc*WSD1

*At*WSD3

*At*WSD2

Clade A

**Supplementary Figure S2. Phylogenetic analysis of bifunctional wax synthase/diacylglycerol acyl transferases (WSD) in *P. trichocarpa* and *Arabidopsis thaliana*.** Clade A was assigned according to Cheng *et al.*, (2022). *A*. *thaliana* WSD amino acids sequences (AGI numbers from: King *et al.*, 2007) were retrieved from the TAIR database (version 1, accessed on 11-May-2023). The *A. thaliana* WSD amino acid were used to extract the amino acid sequences of the *P*. *trichocarpa WSDs* from <https://plantgenie.org> (accessed on 11-May-2023). The phylogenetic tree was built with Geneious Prime (Biomatters, Ltd., Auckland, New Zealand, <https://www.geneious.com>) by global alignment with free ends gaps alignment type and a cost matrix of 70% similarity (International Union of Biochemistry (IUB) nucleotide ambiguity code, 5.0/-4.5). The genetic distance model Jukes-Cantor with Neighbor-joining tree build method was used with the Jojoba wax ester synthase as the outgroup.





**Supplementary Figure S3.** **Expression of Pc*WSD1* in the wild type and of Sc*WS* in the transgenic lines in leaves (L), wood (W), bark (B) and developing xylem (D).** Data show means of n = 4 individual *P*. x *canescens* plants per line (±SE).

**
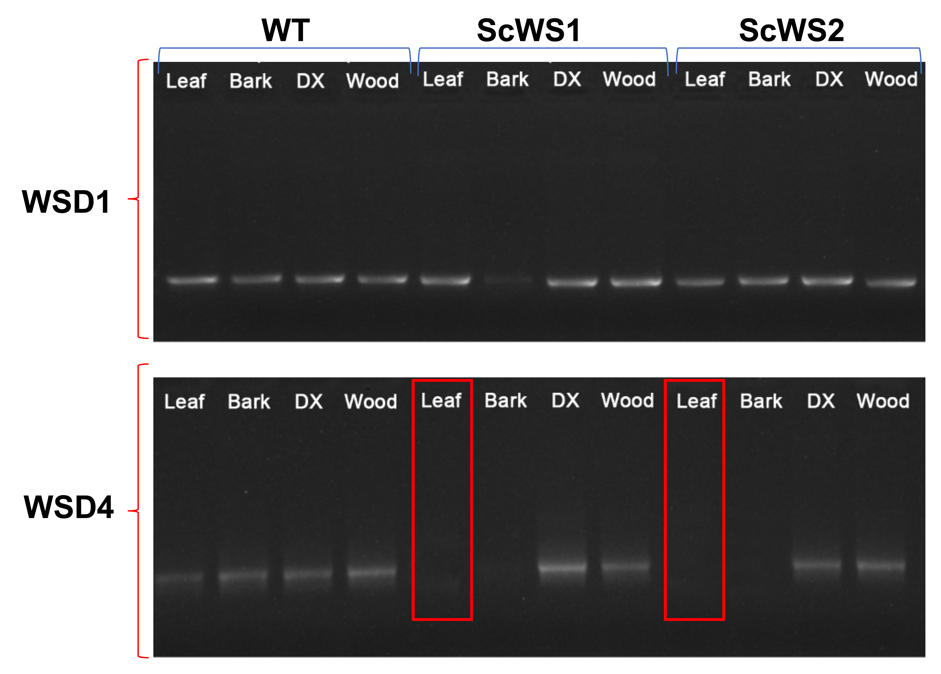
**

**Supplementary Figure S4.** **Expression of *PcWSD1* and *PcWSD4* in different tissues of *P.* x *canescens.*** RNA was extracted from leaves, bark, developing xylem (DX), and wood of wild type (WT) poplar and the lines *Sc*WS1 and *Sc*WS2. RT-PCR was conducted with 10 µl of 70 ng/µl cDNA per slot and the specific primers for *WSD1* and *WSD4* (Supplement Table S3). The products were separated by agarose gel electrophoresis and observed after ethidium bromide staining.

***P.*x *canescens*** **(WT)**

***Sc*WS1**

***Sc*WS2**


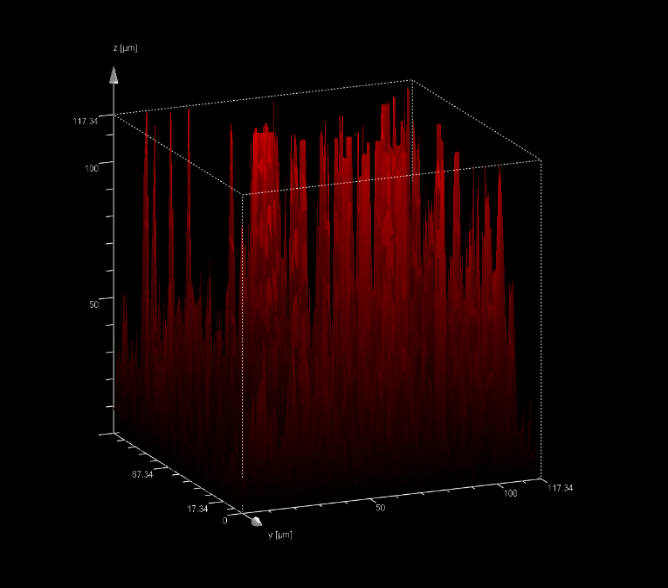

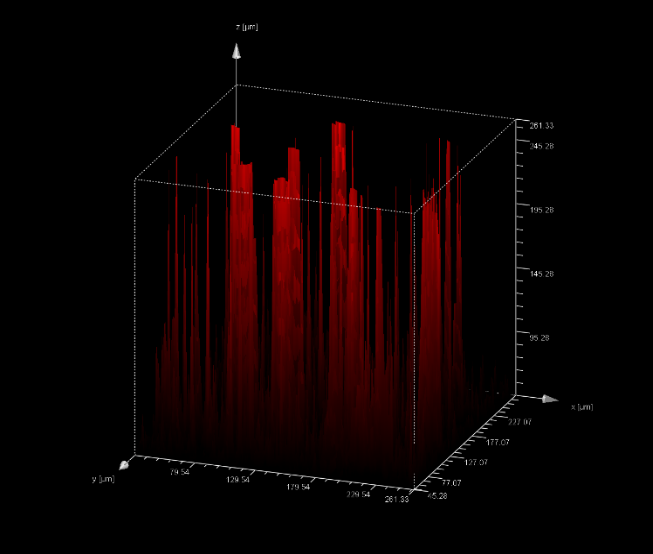

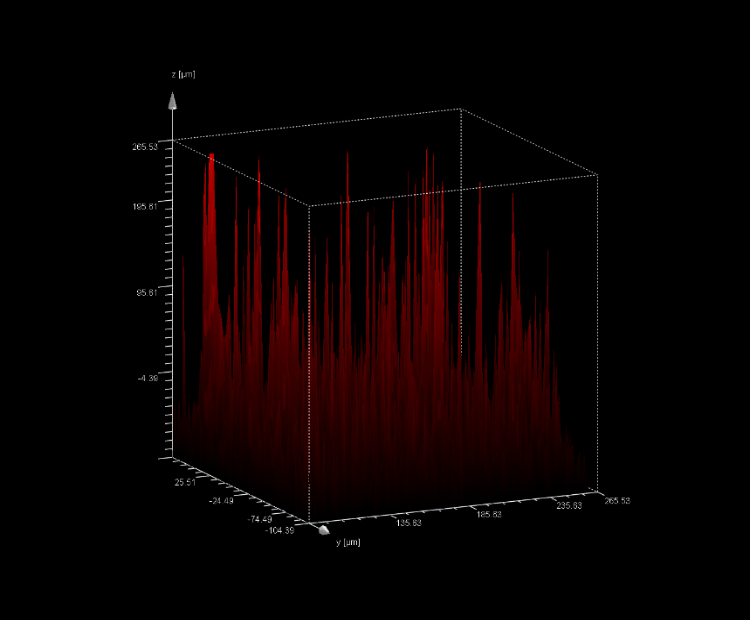


Abaxial side

Adaxial side

**Supplementary Figure S5.** **Confocal laser scanning fluorescence microscopy through the plane of leaves of *P.* x *canescens* wild type and the transgenic lines ScWS1 and ScWS2.** Leaf sections were stained with the lipophilic dye Lipid spot II. Images show examples in 3D views.


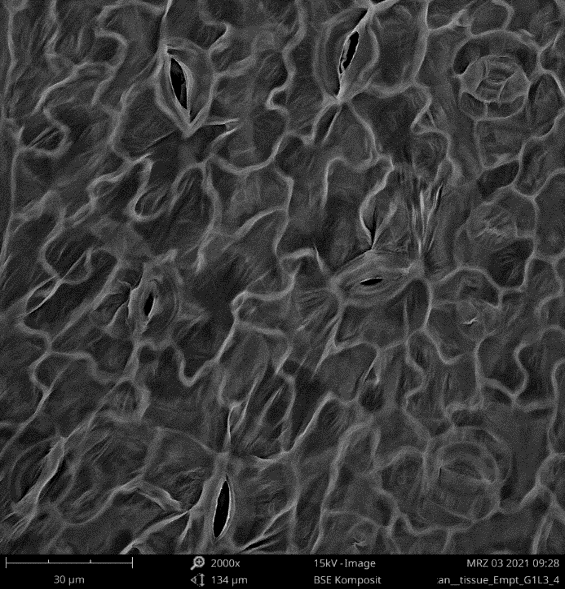

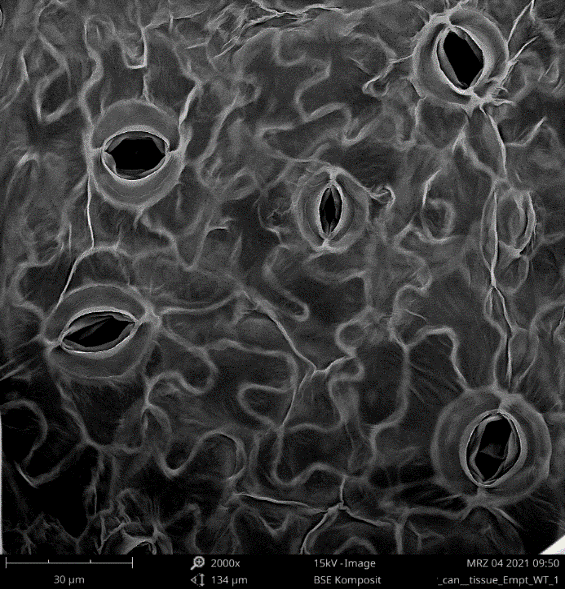

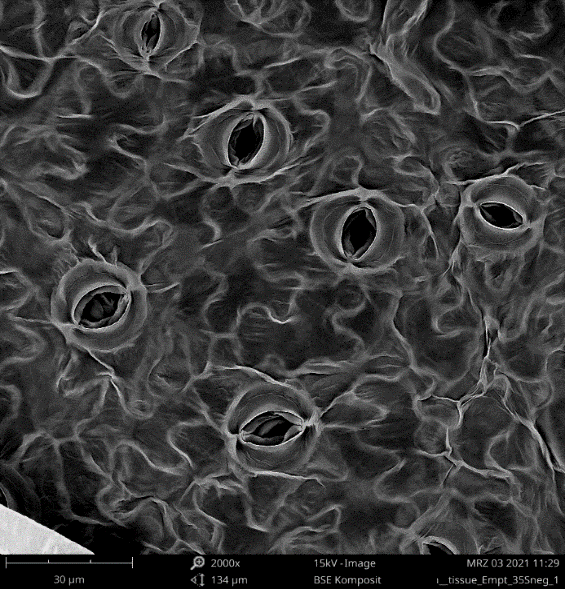


**ScWS1**

**WT (*P*. x *canescens*)**

**pB7WG Vector+35S Promoter**

**(a)**

**(b)**

**(c)**

**

**

**(d)**

**(e)**

**Supplementary Figure S6. Morphology of stomata and stomatal lengths of wild type and transgenic *P*. x *canescens* lines under high humidity.** The plantlets were grown in Magenta boxes under high humidity. Scanning electron microscopy of the abaxial leaf surface (a) wild type, (b) line ScWS1, (c) empty vector control transformed with the *35S* promoter; magnification 2000X. (d) Stomatal lengths, (e) plants in tissue culture. Red arrow indicates the leaf used for the scanning electron microscopy. Data show means (± SE, n = 4 per line). Different letters indicate significant differences among the lines at *P* ≤ 0.05 (post hoc Tukey test).

**
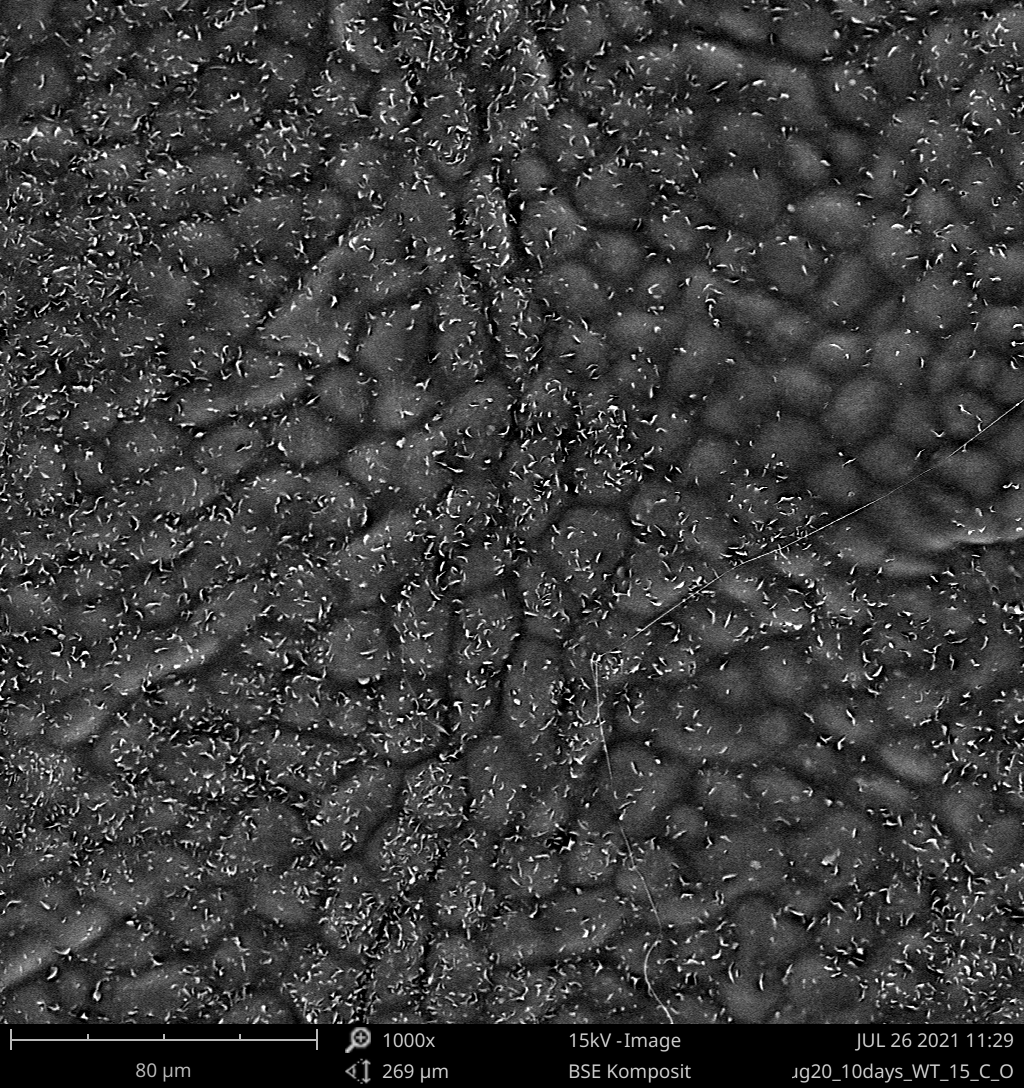

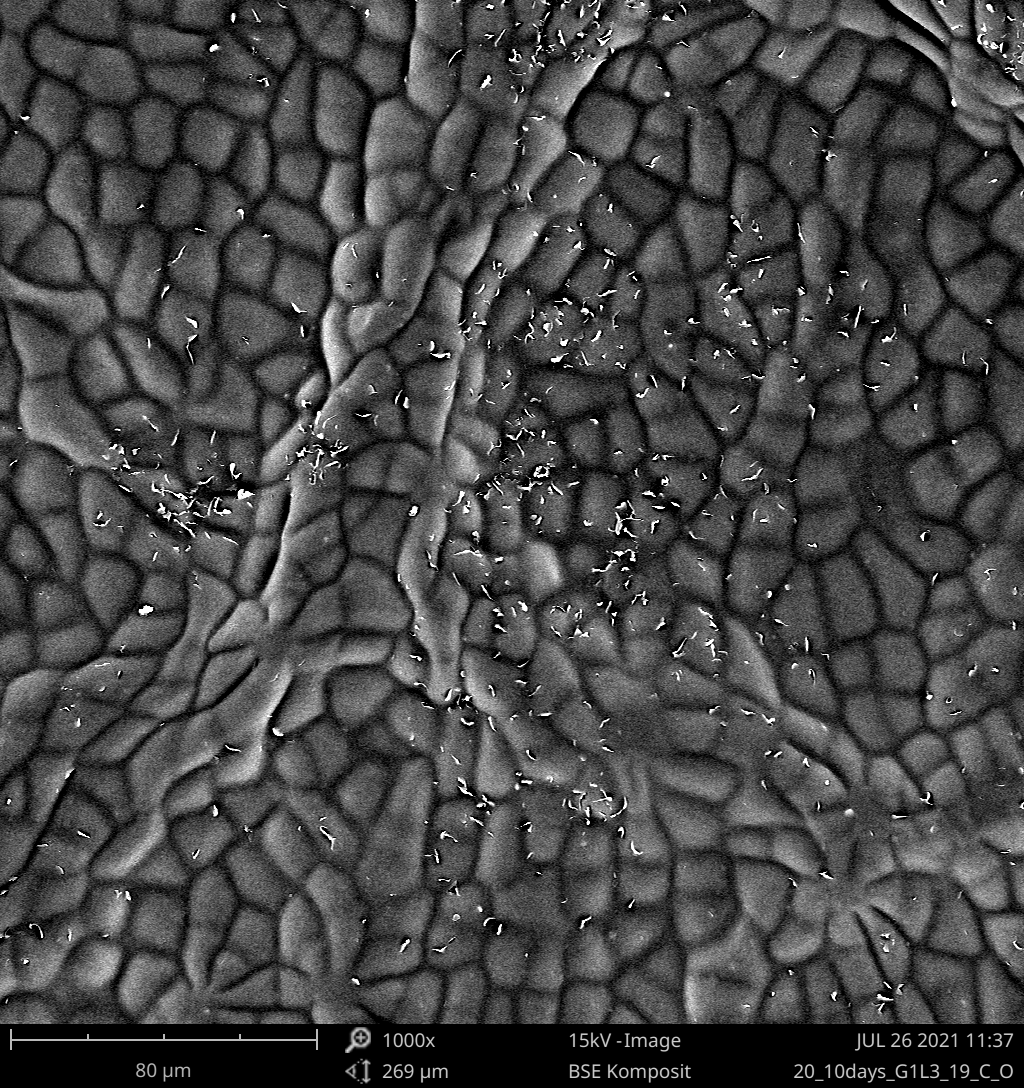
**

(a)

(b)

(c)

80 µm

80 µm

80 µm

**
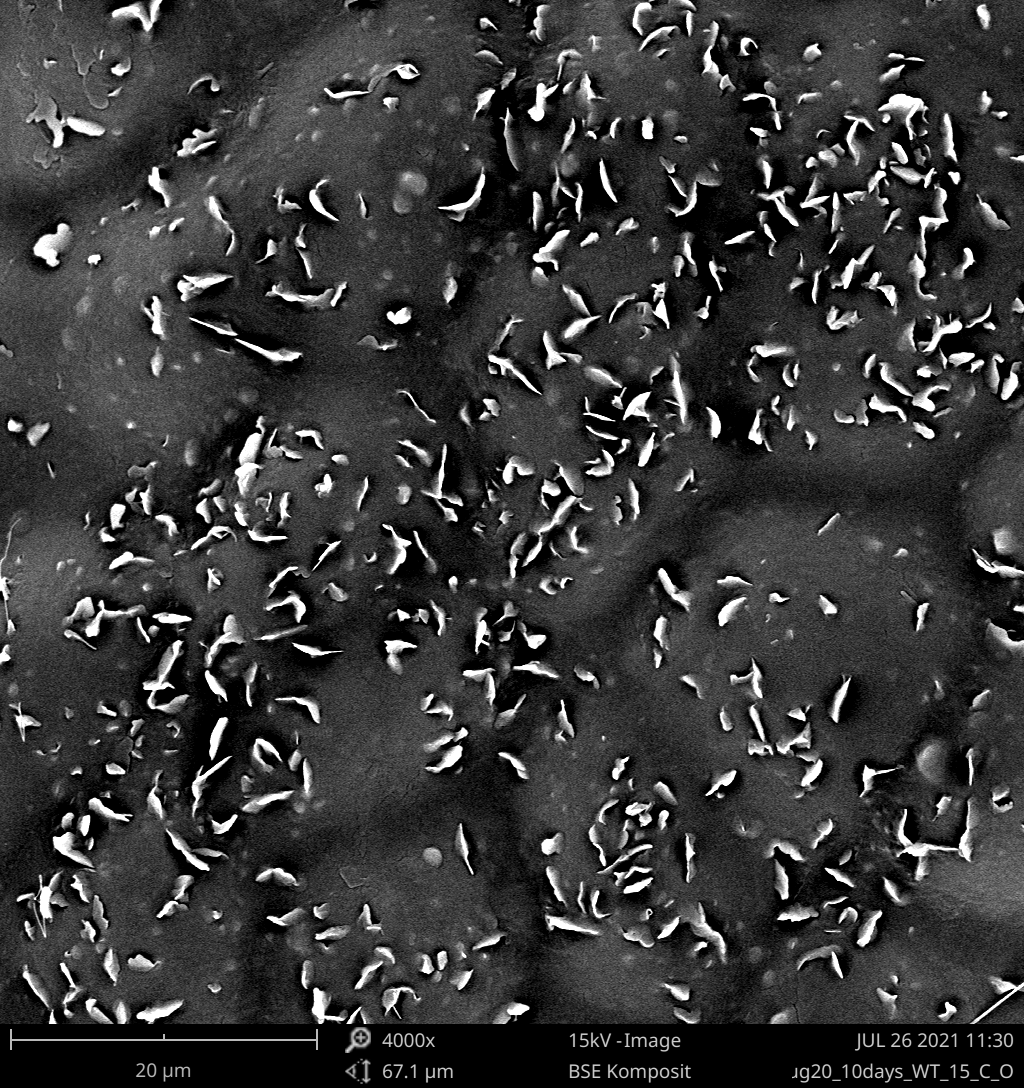

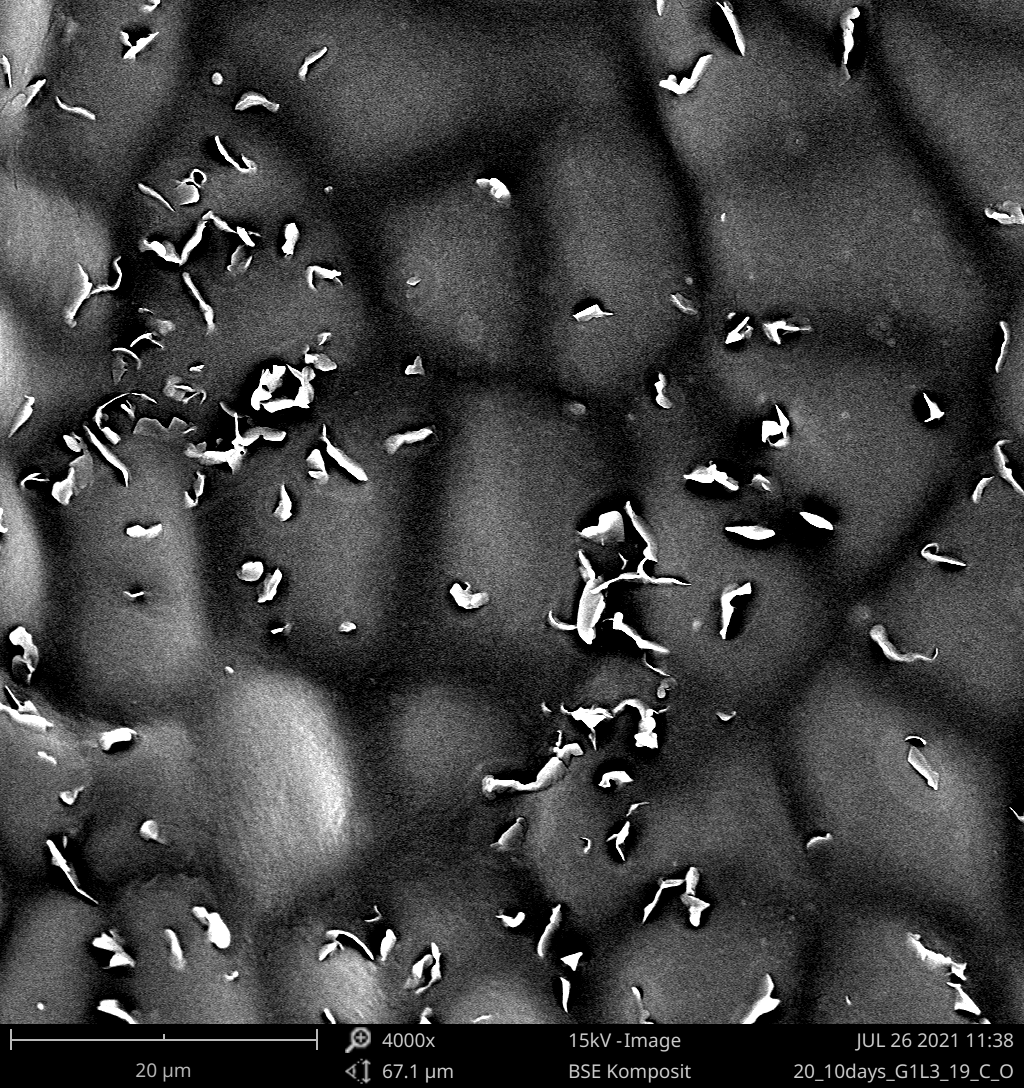
**

(d)

(e)

(f)

20 µm

20 µm

20 µm



**

**



(i)

(h)

(g)

T

80µm

80µm

80µm

**





**

(l)

(k)

(j)

BT

S

CRS

30µm

30µm

30µm

***Sc*WS2**

***Sc*WS1**

***P*. x *canescens* (WT)**

**Supplementary Figure S7**. **Scanning electron microscopic images of the adaxial (a, b, c, d, e, f) and abaxial (g, h, i, j, k, l) leaf surfaces of *P*. x *canescens* wild type (WT) and *Sc*WS lines.** The surfaces are shown at two magnifications: 1000x (a,b,c,g,h,i) and 4000x (d,e,f), and 2000x (j,k,l) The images highlight wax crystals on the adaxial surface. The abaxidal surface shows the stomata (S), cuticular ridges (CR), trichomes (T), and broken trichomes (BT).




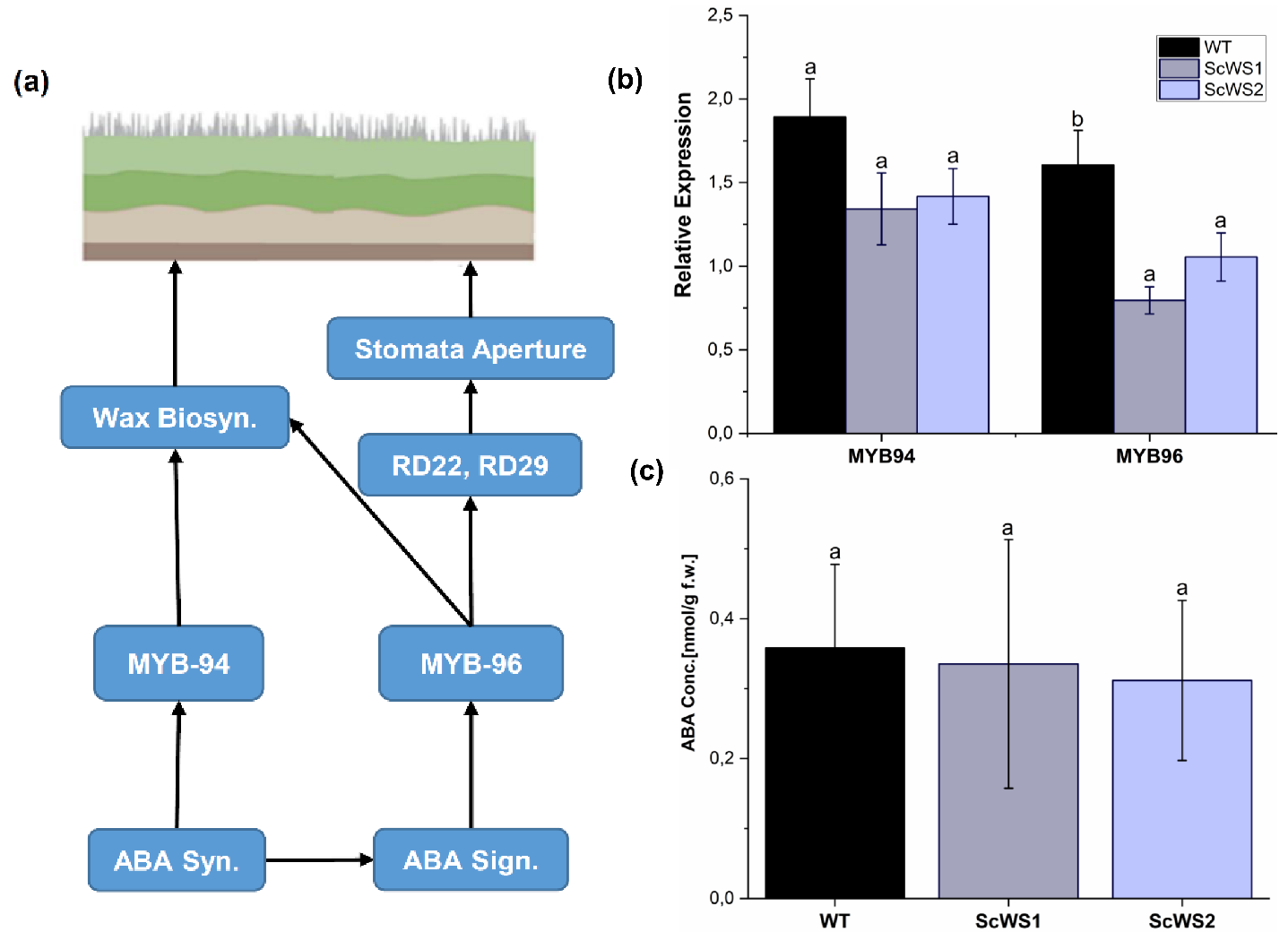


**Supplementary Figure S8**. **Scheme for the** **abscisic acid (ABA) signaling pathway in relation to wax biosynthesis and stoma formation (a),** **relative expression of *MYB 96* and *MYB 94* (b) and ABA concentrations in leaves of wild type and *Sc*WS expressing *P*. x *canescens* plants (c)**. The scheme was adapted from previous publications (Lee *et al.*, 2020; Lewandowska *et al.*, 2020; Seo and Park, 2010; Seo *et al.*, 2009). Abscisic acid was extracted from frozen leaf powder and determined by liquid chromatography coupled with mass spectroscopy as described by Yu *et al.* (2021). Data show means (± SE, n = 4-7 per line). Different letters indicate significant differences at p < 0.05 among the *Sc*WS lines and WT (post hoc Tukey test).


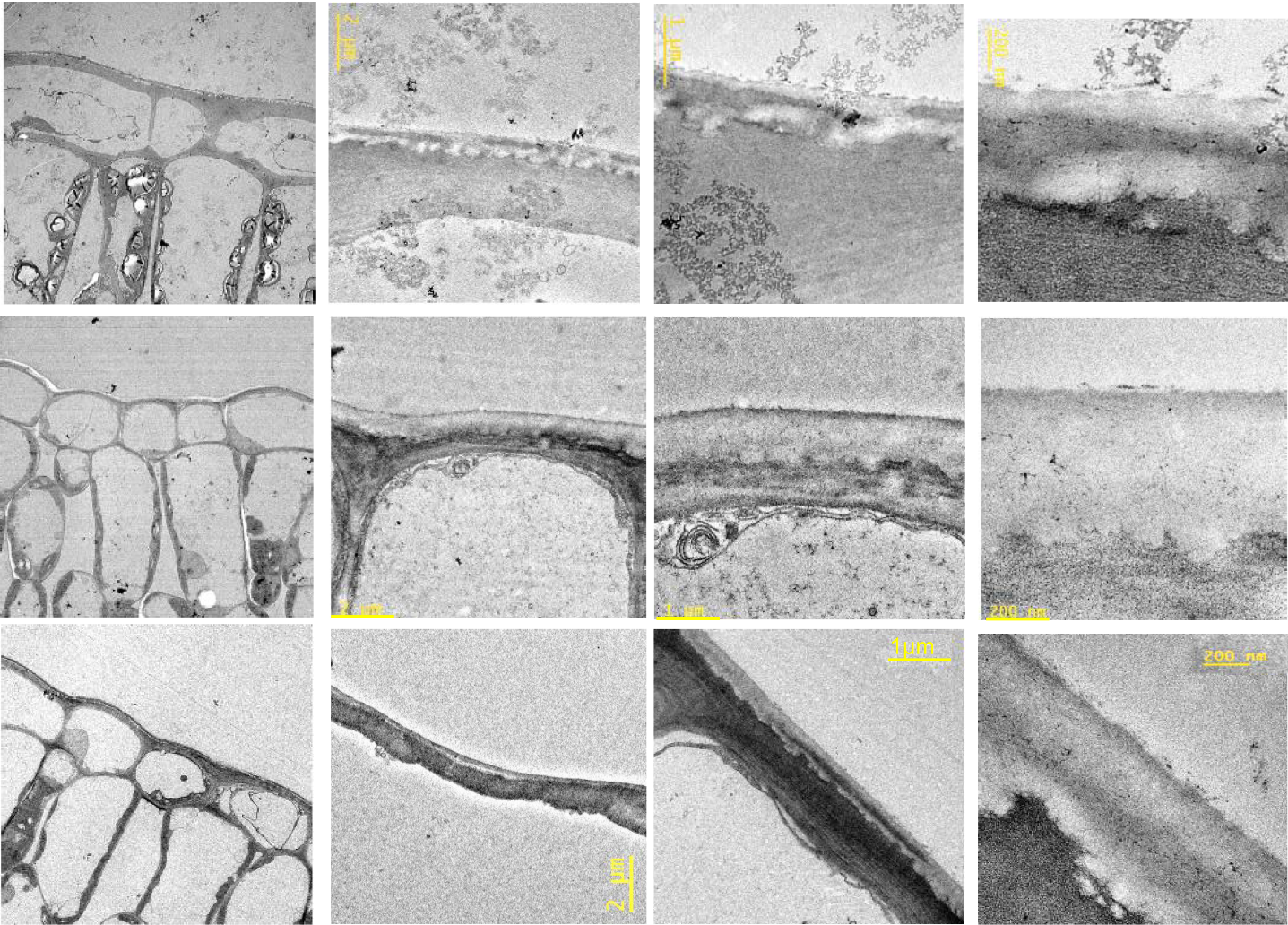


**WT**

***Sc*WS1**

***Sc*WS2**

**Supplementary Figure S9. Transmission electron microscopy of cross sections of *P.* x *canescens* leaves of the wild type and *Sc*WS lines (*Sc*WS1, *Sc*WS2)**. Pictures show the thickness of the cuticle layer with increasing magnification.


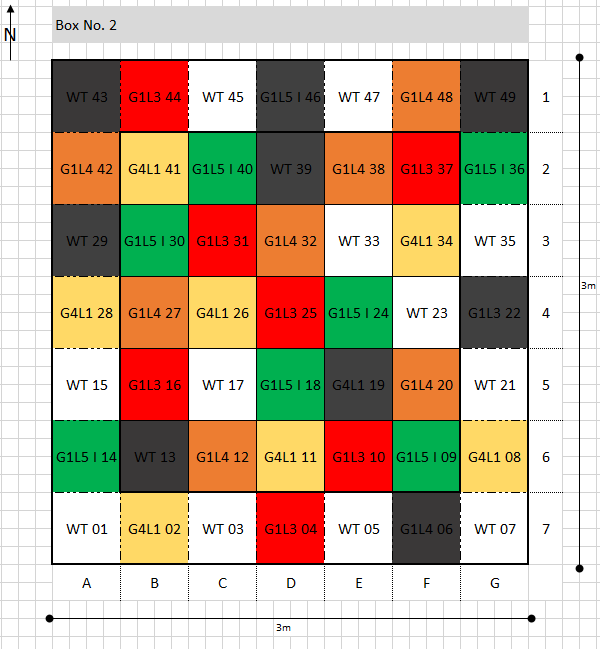


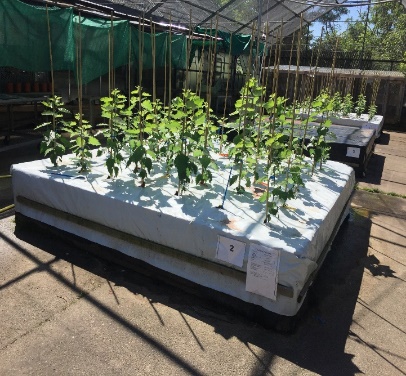


**(c)**

**(a)**

**(b)**


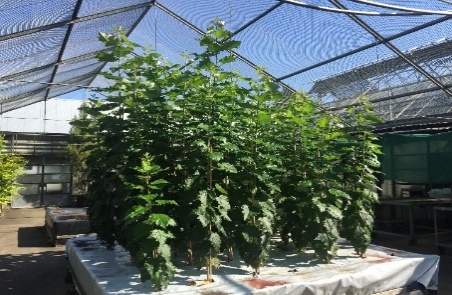


**(d)**


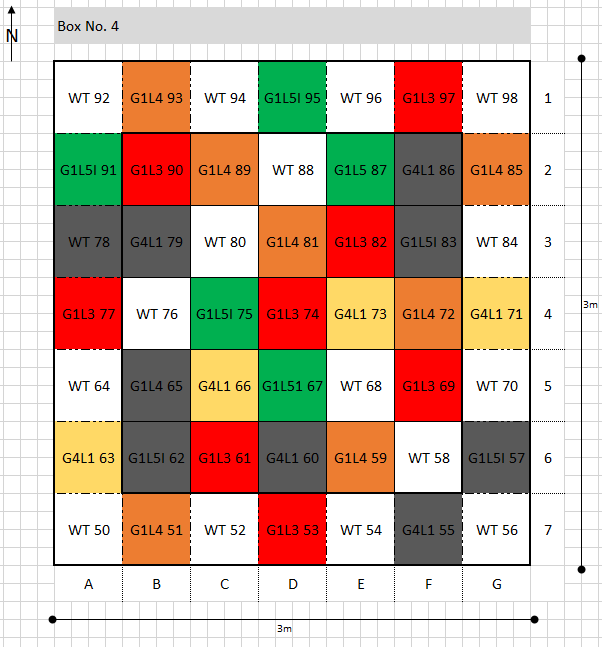


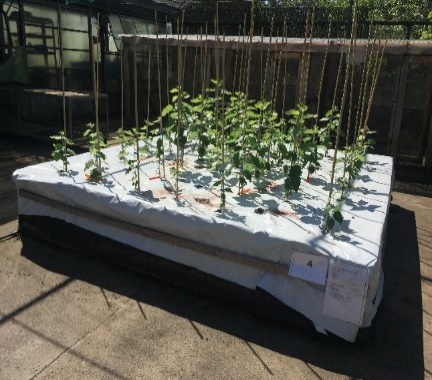


**(e)**

**(b)**

**(e)**


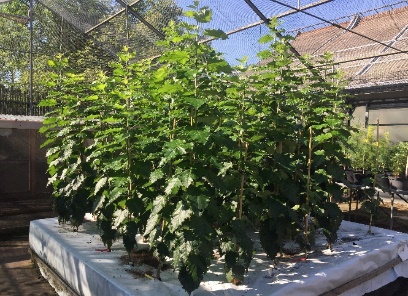


**(f)**

**Supplementary Figure S10.** **Planting scheme of wild type and transgenic *ScWS* expressing poplars (*Populus* × *canescens*) in mixtures under outdoor conditions** (a, b). The poplars were planted in fall 2018. The photos show the plants when the morphological and physiological measurements started in May 2019 (c,e) and when drought treatments started in August 2019 (d,f). Lines used in this experiment, *Sc*WS1 (G1L3), *Sc*WS2 (G1L5.I), *Sc*WS3 (G1L4), *Sc*WS4 (G4L1) and wild type (WT).


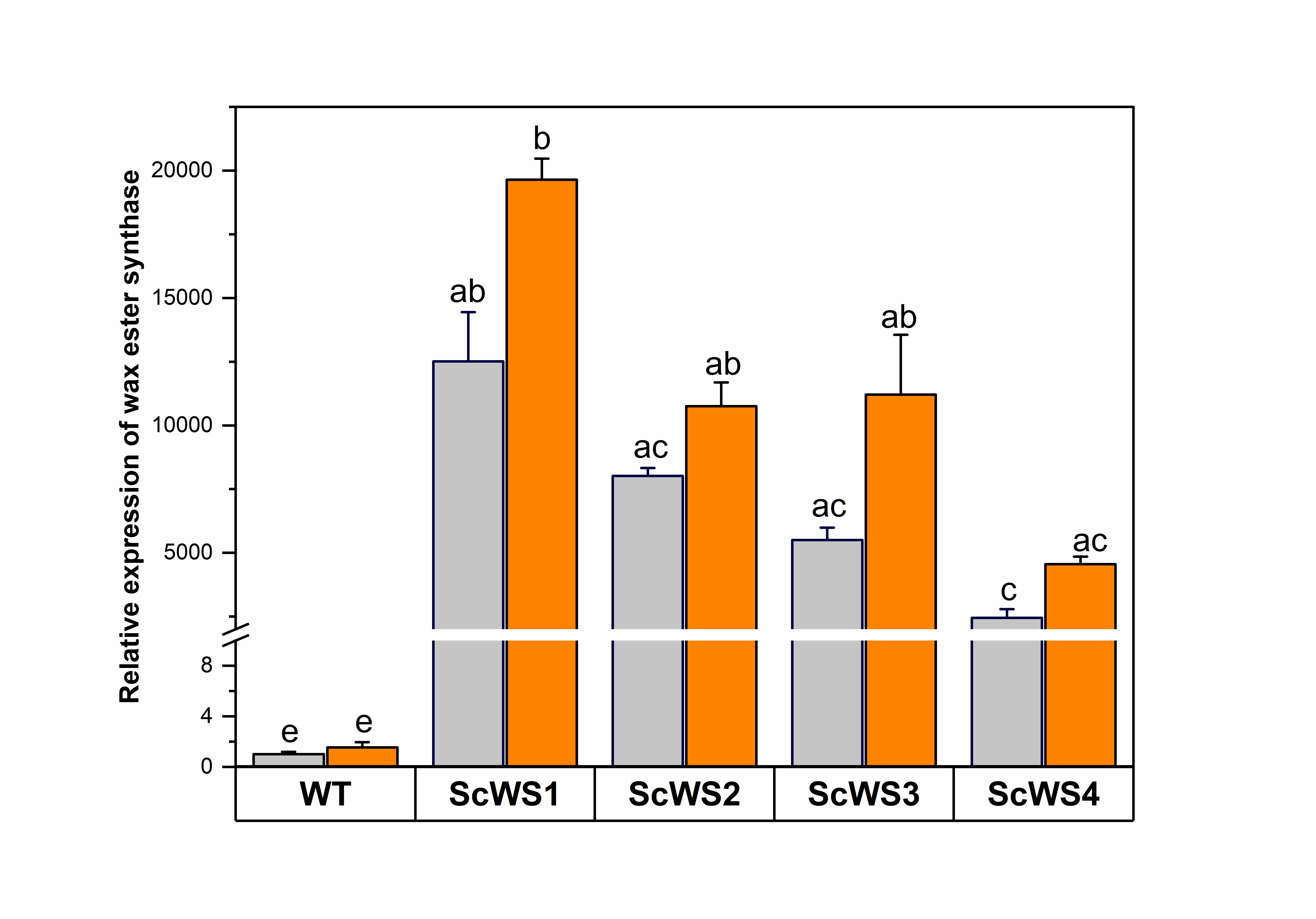


**Supplementary Figure S11.** **Expression of Pc*WDS1* in wild type (WT) and of *ScWS* in transgenic *P*. x *canescens* under outdoor conditions**. The expression levels were determined with primers for *ScWS* and for wax ester synthase/diacylglycerol acyltransferase (Pc*WSD1*) (cf. Supplement Table S3). Grey bars: well-irrigated plants, orange bars: drought-stressed plants. Leaf number five from the top was harvested on 13^th^ September 2019, 6 weeks after drought exposure and used for the analysis. Data show means (± SE, n = 4 plants per line and treatment). Different letters indicate significant differences among the treatments and lines at P ≤ 0.05 (post hoc Tukey test).





**Supplementary Figure S12. Light response curves of photosynthesis of *Sc*WS lines and wild type *P*. x *canescens***. Data show means (± SE, n = 3 independent plants per line). PAR, photosynthetically active radiation. Stars denote significant differences: ∗ P ≤ 0.05; ∗∗P ≤ 0.01 of the WT compared with the *Sc*WS lines. PAR was set to the indicated level and photosynthesis measurements started when the readings were stable (usually after 2 min).

**Supplementary Table S1**. List of the primers and Potri numbers for genes used for the cloning (1,2) and for expression analyses by qRT PCR (3). For cloning, primers were designed with 58°C melting temperature using Geneious Prime (Biomatters, Ltd., Auckland, New Zealand, https://www.geneious.com). For qRT PCR, primers were designed with the PerlPrimer software version 1.1.20 (https://perlprimer.sourceforge.net, open-source PCR primer design) with maximum of 150 bp and 58-62 °C annealing temperature. PtrPPR_2 and PtrRpp14 are the housekeeping genes used for the normalization in the qRT PCR experiments.

|  | Gene name | Potri Nr. | Sequence |
| --- | --- | --- | --- |
| 1 | *Sc*WS | Cloning | FOR: attL1- GGGGACAAGTTTGTACAAAAAAGCAGGCTTAGTCGACATGGAGGTGGAG |
|  |  |  | REV: attL2- GGGGACCACTTTGTACAAGAAAGCTGGGTTCTCACCACCCCAACAAACC |
| 2 | *p*DONR201 | Cloning | FOR: TCGCGTTAACGCTAGCATGGATCTC |
|  |  |  | REV GTAACATCAGAGATTTTGAGACAC |
| 3 | *Sc*WS |  | FOR: ATG GTG GTG AAG AAG GCG G |
|  |  |  | REV: TCC AGT CAC CAT CAC GAA CC |
|  | CER1 | Potri.014G152300 | FOR: TCTTCATCACCACTATCTCTACTC |
|  |  |  | REV: GAATCACAGAAGTAATGGGCTC |
|  | CER2 | Potri.001G319200 | FOR: GTCTTTGTTCAGTTCACTTGGT |
|  |  |  | REV: ATGTGTTGATGAAGGTTGAAGC |
|  | CER4 | Potri.004G185000 | FOR: GAATAGTTGATGTGATACCAGCAG |
|  |  |  | REV: CACGGGATTTCTCACAGAGG |
|  | CER6 | Potri.008G120300 | FOR: GCACTGGTACTTGTTCAAACTC |
|  |  |  | REV: AATTTGTTCAGATACAGGGAGG |
|  | WSD1 | Potri.001G304600 | FOR: TCCATGCAGGATCTATCCGA |
|  |  |  | REV: GATATGATACGCGAAGTGAGCC |
|  | WSD4 | Potri.014G098900 | FOR: TTTATCTGTAGTCATCATACGCCC |
|  |  |  | REV: TGCTGGTATCACATCAACTATTCC |
|  | OSP1 | Potri.014G160100 | FOR: GTGATGTGCTATCTGTGATACC |
|  |  |  | REV: CCTATGCTTATCATGTGTATACCC |
|  | MYB96 | Potri.004G126700 | FOR: AAAGCAAGGGTAGTGTTCTTACTC |
|  |  |  | REV: CTAAGCAAACCTGTATTAGTAGGC |
|  | MYB94 | Potri.017G082500 | FOR: CACCTTGCTGTGATAAGATAGGA |
|  |  |  | REV: TCTAAGCAGTCCTGTACTAGTTGG |
|  | *ABCG32* | Potri.001G311300 | FOR: TGGCTATAATGGGTCCTTCAG |
|  |  |  | REV: GAGTTACATAAGCCGATGTACC |
|  | *LPTG* | Potri.009G158100 | FOR: AACACCTCAAGGTCCATCTC |
|  |  |  | REV: GGAGAAATGAAGATGCTAAGCC |
|  | *Ptr*PPR_2 | Potri.012G141400 | FOR: ATCGTTCCAAGTCAAGTATGTG |
|  |  |  | REV: TCAAGGGAGCAACTTTACAG |
|  | *Ptr*Rpp14 | Potri.015G001600 | FOR: GCAATGTGAGGAGTTTAGGG |
|  |  |  | REV: TATTAAATGTCTGTGCTGTAGTGTG |

**Supplementary Table S2:** **Night respiration, transpiration and stomatal conductance of well-irrigated and drought-stressed *Sc*WS lines and wild type *P*. x *canescens* in a long-term greenhouse experiment**. The measurements were performed at night under the following conditions: air temperature 21.0 ± 0.5 °C, air humidity 70 % ± 13 %, ambient CO_2_ of 419 ± 1 ppm and photosynthetic photon flux at leaf level 0 μmol m^-2^ s^-1^. Measurements were conducted 12 days after the start of the drought treatment. Data show means (±SE, n = 4). Two-way ANOVA with line and treatment as main factors was used to compare differences of means among poplar lines and drought, followed by a posthoc test (Tukey). Means that differ at p ≤ 0.05 are indicated by different letters.

| Line | Treatment | Respiration  (µmol m^-2^ s ^-1^) | SE |  | Stomatal conductance  (mol m^-2^ s ^-1^) | SE |  | Transpiration  (mmol m^-2^ s ^-1^) | SE |  |
| --- | --- | --- | --- | --- | --- | --- | --- | --- | --- | --- |
| ScWS-1 | **Control** | -1.048 | 0.10 | ab | 0.06 | 0.019 | a | 1.73 | 0.53 | a |
| ScWS-2 | **Control** | -1.04 | 0.15 | ab | 0.038 | 0.012 | ab | 1.178 | 0.33 | ab |
| WT | **Control** | -1.28 | 0.27 | a | 0.029 | 0.0083 | a | 0.907 | 0.24 | bc |
| ScWS-1 | **Drought** | -0.75 | 0.12 | b | 0.017 | 0.004 | b | 0.55 | 0.11 | bc |
| ScWS-2 | **Drought** | -0.76 | 0.21 | b | 0.016 | 0.0042 | b | 0.53 | 0.12 | bc |
| WT | **Drought** | -1.16 | 0.17 | ab | 0.012 | 0.0002 | b | 0.413 | 0.004 | c |

**Supplementary Table S3:** **Night respiration, transpiration and stomatal conductance of well-irrigated and drought-stressed *Sc*WS lines and wild type *P*. x *canescens* under field conditions**. The measurements were performed at night under the following conditions: air temperature 18.0 ± 0.3 °C, air humidity 57.9 % ± 2.1 %, ambient CO_2_ 395.6 vpm and photosynthetic photon flux at leaf level 0 μmol m^-2^ s^-1^. Measurements were conducted 4 weeks after the start of the drought treatment. Data show means (± SE, n = 4). Two-way ANOVA with line and treatment as main factors was used to compare differences of means among poplar lines and drought, followed by a posthoc test (Tukey). Means that differ at p ≤ 0.05 are indicated by different letters.

| Line | Treatment | Respiration  (µmol m^-2^ s ^-1^) | SE |  | Stomatal conductance  (mol m^-2^ s ^-1^) | SE |  | Transpiration  (mmol m^-2^ s ^-1^) | SE |  |
| --- | --- | --- | --- | --- | --- | --- | --- | --- | --- | --- |
| ScWS-1 | **Control** | -0.207 | 0.13 | ab | 0.04 | 0.015 | a | 5.93 | 1.29 | ab |
| ScWS-2 | **Control** | -0.34 | 0.20 | ab | 0.046 | 0.013 | ab | 5.84 | 1.82 | ab |
| ScWS-3 | **Control** | -0.23 | 0.03 | ab | 0.045 | 0.0097 | a | 5.12 | 0.51 | ab |
| ScWS-4 | **Control** | -0.28 | 0.09 | ab | 0.04 | 0.009 | ab | 5.86 | 0.31 | ab |
| WT | **Control** | -0.44 | 0.25 | a | 0.09 | 0.013 | c | 7.76 | 0.34 | c |
| ScWS-1 | **Drought** | -0.13 | 0.01 | b | 0.033 | 0.011 | a | 4.14 | 0.69 | ab |
| ScWS-2 | **Drought** | -0.11 | 0.06 | b | 0.044 | 0.02 | a | 5.76 | 1.13 | ab |
| ScWS-3 | **Drought** | -0.11 | 0.15 | b | 0.055 | 0.01 | ac | 7.22 | 1.14 | a |
| ScWS-4 | **Drought** | -0.1 | 0.11 | b | 0.061 | 0.016 | ab | 7..22 | 0.22 | a |
| WT | **Drought** | -0.13 | 0.03 | b | 0.087 | 0.02 | bc | 5.55 | 1.45 | b |

**Supplementary Table S4.** **Gas exchange of *P*. x *canescens* wild type and *Sc*WS lines under outdoor conditions in the second growth year.** Gas change measurements were conducted in July 2020 using LICOR 6800, from 10am to 3pm. n = 5 per line per treatment. The average air temperature and ambient humidity during the measurement were 23.5 ± 0.3°C and 58% ± 2% and the photon flux density of the photosynthetically active radiation 800 μmol m^−2^ s^−1^ at the leaf surface. Water use efficiency (WUE) was calculated as the ratio of photosynthetic rate to transpiration rate.

| Line | Photosynthesis  (µmol m^-2^ s ^-1^) | SE |  | Stomatal conductance  (mmol m^-2^ s ^-1^) | SE |  | Transpiration (mmol m^-2^ s ^-1^) | SE |  | WUE  (µmol mol^-1^) | SE |  |
| --- | --- | --- | --- | --- | --- | --- | --- | --- | --- | --- | --- | --- |
| ScWS-1 | 12.01 | 2.47 | ab | 200 | 95 | a | 2.86 | 1.04 | a | 5218.45 | 2651.24 | b |
| ScWS-2 | 15.51 | 2.93 | ab | 350 | 115 | a | 4.01 | 1.05 | a | 4263.76 | 1717.42 | b |
| ScWS-3 | 15.11 | 3.57 | ab | 250 | 118 | a | 3.86 | 0.99 | a | 3032.79 | 1150.63 | ab |
| ScWS-4 | 10.87 | 2.37 | b | 410 | 113 | a | 4.73 | 1.06 | a | 3509.13 | 1173.54 | ab |
| WT | 20.79 | 3.69 | ab | 1210 | 200 | b | 6.35 | 1.07 | b | 3585.95 | 1352.22 | a |

**Supporting Methods S1: Protocols for poplar transformation, scanning electron microscopy of fresh leaf surfaces and cuticular wax analysis**

**Poplar transformation protocol**

The *Sc*WS clone was obtained from Prof. I Feussner (Department for Plant Biochemistry, University of Göttingen, Göttingen, Germany). Primers suitable for the Gateway system™ (Biomatters, Ltd., Auckland, New Zealand, <https://www.geneious.com>) were used for amplification of the vector (Supplement Table S5). The PCR products were confirmed by electrophoresis, purified using the innuPREP PCRpure Kit (analytik jena AG, Jena, Germany) and cloned into the pDONOR-201 vector (Thermo Fisher Scientific, Waltham, MA, USA) containing the p*35S* promoter to create the gateway pEntry clone. To obtain the binary vector, pEntry::p*35S*::*Sc*WS was cloned into pK7WG using the Gateway system (Invitrogen, Waltham, Massachusetts, USA) and used to transform *Agrobacterium tumefaciens* strain GV3101 pMP90 (BacDive, Braunschweig, Germany). For this purpose, aliquots of competent Agrobacteria (200 µl) were thawed in an ice bath. Then, 1 µl, which contained approximately 100 to 1000 ng of the binary vector DNA, was added to the Agrobacteria and incubated on ice for 5 minutes with occasional stirring. Subsequently, the suspension was frozen in liquid nitrogen for 3 minutes, thawed in a water bath at 37°C and further incubated at 37°C for 5 minutes. Then, 800 µl of YEB (yeast extract broth) medium (without antibiotics) was added the suspension was incubated at 28°C on a shaker (Eppendorf, Köln, Germany) for 2 hours. The suspension was then centrifuged for 2 minutes at 5000 rpm at room temperature. Subsequently, the cells were re-suspended in YEB and plated onto two Petri dishes, containing YEB agar supplemented with the appropriate antibiotic as required.

Agrobacteria transformed with the binary vector were grown over night in 4 ml YEB – medium (with following ingredient: beef extract 5g/l, yeast extract 1g/l, peptone 5g/l, sucrose 5g/l, MgSO_4_ 0.3 g/l, and agar 20 g/l, pH 7.2) containing antibiotics (Rifampicin, 50 μg ml^-1^; Gentamycin, 25 μg ml^-1^) at 28°C in darkness on an orbital shaking incubator (Sanyo Gallenkamp PLC, Cambridge, United Kingdom) at 90 rpm to generate the starter culture. The starter culture (2 ml) was used to inoculate 100 ml YEB medium (without antibiotics) at 28°C. The culture was grown in darkness at 28°C on a shaker at 90 rpm for about 3 to 5 hours to reach an OD_600_ of 0.3 to 0.5. Then, 20 µM 3´,5´-dimethoxy-4´-hydroxy acetophenone (Sigma Aldrich, Darmstadt, Germany) was added and the incubation was continued for another 30 min.

To transform *P*. x *canescens*, 4-week-old plantlets from a tissue culture were used. The leaves of the plantlets were removed. The stem was divided into small segments of 1 to 2 cm length and transferred into the suspension with the transformed Agrobacteria. The stem segments were incubated in darkness at 28°C for 30 min on a shaker (120 rpm). The stem sections were strained, drained, and transferred to agar plates containing ½ MS (Murashige and Skoog, 1962) medium supplemented with 7g L^-1^ plant agar, 20 g L^-1^ sucrose and were kept in darkness at 25°C for one week. The stem sections, overgrown with Agrobacteria, were washed three times in sterile distilled water with 400 µg ml^-1^ ticarcillin clavulanate (Duchefa, Haarlem, Netherland) for three minutes in an Erlenmeyer flask. Sterile tap water without ticarcillin clavulanate was used for the last washing step.

To screen for the positively transformed plantlets, the stem sections were transferred into selection medium (½MS medium supplemented with 20 g l^-1^ sucrose, and 7 g l-^1^ plant agar, pH 5.8 and the following antibiotica: Pluronic F-68 (0.01%), Thidiazuron (0.0022 mg l^-1^), Cefotaxim (150 mg l^-1^), Timentin (150 mg l^-1^), Kanamycin (50 mg l^-1^) (Duchefa, Haarlem, Netherland). The stem sections were kept for four to six weeks under long day conditions with 16 h of low light, approximately 10 µmol photons m^-2^ s^-1^ photosynthetic active radiation (PAR) (fluorescent lamps L18W/840, Osram, Munich, Germany) at 22°C and 20 to 40 % humidity. When shoots emerged, the plant culture was transferred to rooting medium (½ MS medium, 20 g l^-1^ sucrose, and 7 g l^-1^ plant agar, pH 5.8 with the selection antibiotic Kanamycin (50 mg l^-1^) and incubated under the same conditions above but with 20 µmol photons m^-2^ s^-1^ PAR. Roots produced within five to eight weeks indicated successful transformation. The rooted shoots were grouped in lines and transferred to glass jars (350 ml) with rooting medium with Kanamycin (50 mg l^-1^). Regenerating plantlets from independent transformation events were tested for the presence of the target gene *Sc*WS by Sanger sequencing (Microsynth Seqlab, Göttingen, Germany) after extracting the genomic DNA of one leaf per line (Edwards *et al.*, 1991). Successfully transformed independent lines carrying the *p*35S::*Sc*WS constructs were denominated ScWS1, ScWS2… ScWSn. The lines were kept separately in tissue culture as described by Müller et al. (2013) and were used as the stock for further experiments.

**Scanning electron microscopy of fresh leaves**

Two rectangular pieces of approximately 1 cm x 1 cm were cut from each leaf with a double edge razor blade (Wilkinson Sword, United Kingdom) and used for inspection of the ad- and abaxial surface as described by Dreischhoff *et al.*, (2023). The specimen was transferred on top of a Standard SEM Pin Stub Mount (diameter 12.7 mm, Plano GmbH, Wetzlar), which had been covered by conductive double-sided adhesive carbon tape (Plano GmbH, Wetzlar, Germany). Immediately afterwards, the sample was covered with a gold layer of 10 nm thickness (Q150R S/E/ES plus sputter coater, Quorum Technologies Ltd, Lewes, United Kingdom). Preliminary tests showed that preceding freeze-drying can be skipped, if the samples were measured within a short period of time (about 2 days). After sputter coating, the edges of the sample were sealed with a conductive carbon cement (Plano GmbH, Wetzlar, Germany). SEM imagine acquisition was performed in the back-scattered electron mode at a voltage of 15kV and a resolution of 1024 pixels with Phenom ProX (G5) desktop SEM (Phenom-World, Eindhoven, Netherlands). Images of two different leaf area were recorded at different magnifications.

**Cuticular wax analyses**

For cuticular wax extraction, three leaf discs of 14 mm in diameter with an area of 2.5 cm² were collected and analyzed as previously described (Haslam and Kunst, 2013). Plant tissues were immersed in chloroform for 30 s containing 2.5 µg of *n*-tetracosane (C24 alkane) (Merck KGaA, Darmstadt, Germany) as an internal standard. Samples were dried under a stream of nitrogen at room temperature, resuspended in 200 µL of chloroform and evaporated, before derivatization with 10 µL N,O-bis(trimethylsilyl) trifluoroacetamide, 1 % chlorotrimethylsilane (Merck KGaA) and 10 µL of pyridine (Merck KGaA) for 1 h at 80 °C. Derivatization reagents were evaporated under nitrogen gas and samples were resuspended in 40 µL chloroform. For quantitative analysis, 2 µL of each sample were injected onto an Agilent 6890 gas chromatograph coupled with flame ionization detection equipped with a DB1-ht column (30 m x 0.32 mm, 0.1 µm coating thickness [Agilent Technologies Deutschland GmbH, Ratingen, Germany]) using a helium carrier gas inlet at a flow rate of 1.2 mL/min. Cuticular wax analysis was performed in a 5:1 split mode. The oven temperature gradient was as follows: 50 °C for 2 min, ramped by 40 °C/min to 200 °C, held at 200 °C for 1 min, increased by 3 °C/min to 320 °C and held at 320 °C for 15 min. The amount of wax was determined by comparing the area of the internal standard using the Agilent ChemStation software and expressed per unit tissue area (cm²).

For peak identification, the identity of the wax components was confirmed via GC coupled with a mass selective detector (GC-MS) equipped with a DB1-ht column (Agilent 5977B mass selective detector connected to an Agilent 7890B GC system). The mass range was set between 50 and 900 amu. The transfer line was set at 280 °C, the ion source at 230 °C, and the electron energy was 70 eV.

**References**

Cheng, D., Li, L., Rizhsky, L., Bhandary, P. and Nikolau, B.J. (2022) Heterologous expression and characterization of plant wax ester producing enzymes. Metabolites 12, 577.

Dreischhoff, S., Das, I.S., Häffner, F., Wolf, A.M., Polle, A. and Kasper, K.H. (2023) Fast and easy bioassay for the necrotizing fungus *Botrytis cinerea* on poplar leaves. Plant Methods 19, 32.

Edwards, K., Johnstone, C. and Thompson, C. (1991) A simple and rapid method for the preparation of plant genomic DNA for PCR analysis. Nucleic Acids Research 19, 1349.

Haslam, T.M. and Kunst, L. (2013) Extending the story of very-long-chain fatty acid elongation. Plant Science 210, 93–107.

King, A., Nam, J.-W., Han, J., Hilliard, J. and Jaworski, J.G. (2007) Cuticular wax biosynthesis in Petunia petals: cloning and characterization of an alcohol-acyltransferase that synthesizes wax-esters. Planta 226, 381–394.

Lee, S.B., Yang, S.U., Pandey, G., Kim, M.S., Hyoung, S., Choi, D., Shin, J.S. and Suh, M.C. (2020) Occurrence of land-plant-specific glycerol-3-phosphate acyltransferases is essential for cuticle formation and gametophore development in *Physcomitrella patens*. New Phytologist 225, 2468–2483.

Lewandowska, M., Keyl, A. and Feussner, I. (2020) Wax biosynthesis in response to danger: its regulation upon abiotic and biotic stress. New Phytologist 227, 698–713.

Müller A, Volmer K, Mishra-Knyrim M, Polle A (2013) Growing poplars for research with and without mycorrhizas. Frontiers in Plant Science 4:322.

Murashige, T. and Skoog, F. (1962) A revised medium for rapid growth and bio assays with tobacco tissue cultures. Physiologia Plantarum 15, 473–497.

Seo, P.J. and Park, C.-M. (2010) MYB96-mediated abscisic acid signals induce pathogen resistance response by promoting salicylic acid biosynthesis in Arabidopsis. New Phytologist 186, 471–483.

Seo, P.J., Xiang, F., Qiao, M., Park, J.-Y., Lee, Y.N., Kim, S.-G., Lee, Y.-H., Park, W.J. and Park, C.-M. (2009) The MYB96 transcription factor mediates abscisic acid signaling during drought stress response in Arabidopsis. Plant Physiology 151, 275–289.

Yu, D., Janz, D., Zienkiewicz, K., Herrfurth, C., Feussner, I., Chen, S. and Polle, A. (2021) Wood formation under severe drought invokes adjustment of the hormonal and transcriptional landscape in poplar. International Journal of Molecular Sciences 22, 9899.
